# Sensor systems of KEAP1 uniquely detecting oxidative and electrophilic stresses separately *In vivo*

**DOI:** 10.1016/j.redox.2024.103355

**Published:** 2024-09-17

**Authors:** Miu Sato, Nahoko Yaguchi, Takuya Iijima, Aki Muramatsu, Liam Baird, Takafumi Suzuki, Masayuki Yamamoto

**Affiliations:** a Departments of Medical Biochemistry, Tohoku University Graduate School of Medicine, 2-1 Seiryo-machi, Aoba-ku, Sendai, 980-8575, Japan; b Department of Biochemistry & Molecular Biology, Tohoku Medical Megabank Organization, Tohoku University, 2-1 Seiryo-machi, Aoba-ku, Sendai, 980-8573, Japan

## Abstract

In the KEAP1-NRF2 stress response system, KEAP1 acts as a sensor for oxidative and electrophilic stresses through formation of S–S bond and C–S bond, respectively. Of the many questions left related to the sensor activity, following three appear important; whether these KEAP1 sensor systems are operating *in vivo*, whether oxidative and electrophilic stresses are sensed by the similar or distinct systems, and how KEAP1 equips highly sensitive mechanisms detecting oxidative and electrophilic stresses *in vivo*. To address these questions, we conducted a series of analyses utilizing KEAP1-cysteine substitution mutant mice, conditional *selenocysteine-tRNA* (*Trsp*) knockout mice, and human cohort whole genome sequence (WGS) data. Firstly, the *Trsp*-knockout provokes severe deficiency of selenoproteins and compensatory activation of NRF2. However, mice lacking homozygously a pair of critical oxidative stress sensor cysteine residues of KEAP1 fail to activate NRF2 in the *Trsp*-knockout livers. Secondly, this study provides evidence for the differential utilization of KEAP1 sensors for oxidative and electrophilic stresses *in vivo*. Thirdly, theoretical calculations show that the KEAP1 dimer equips quite sensitive sensor machinery in which modification of a single molecule of KEAP1 within the dimer is sufficient to affect the activity. WGS examinations of rare variants identified seven non-synonymous variants in the oxidative stress sensors in human KEAP1, while no variant was found in electrophilic sensor cysteine residues, supporting the fail-safe nature of the KEAP1 oxidative stress sensor activity. These results provide valuable information for our understanding how mammals respond to oxidative and electrophilic stresses efficiently.

## Highlights

• KEAP1-Cys226/613 is required for the compensatory activation of NRF2 under selenoprotein deficiency.
• KEAP1 differentially utilizes sensors detecting oxidative and electrophilic stresses *in vivo*.
• KEAP1 homodimer uses sensitive sensors with a single-molecule inactivation mechanism.

## Graphical abstract

**Figure:**
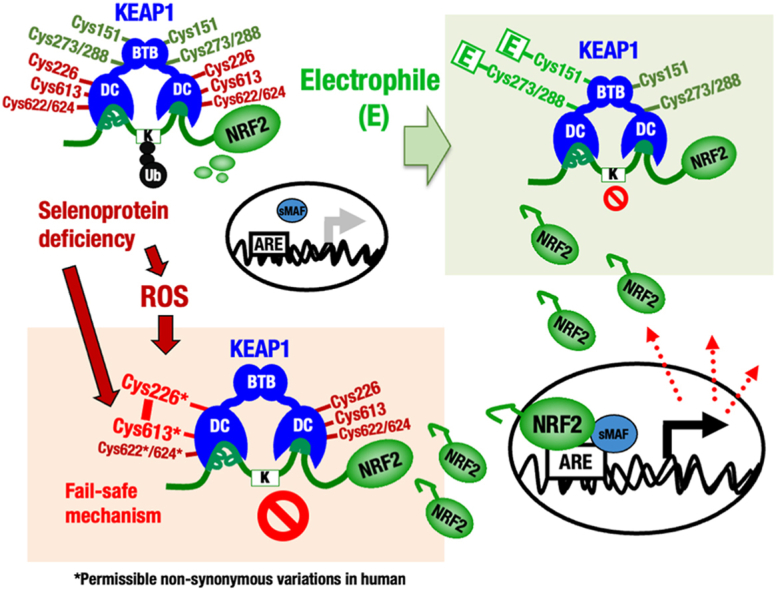

## 1 Introduction

Oxidative stress underlies the development and progression of many diseases, including Alzheimer’s disease, atherosclerosis, and carcinogenesis [1,2]. To overcome this stress, cells are equipped with elaborate defense mechanisms that maintain homeostasis against an ever-changing environment. The transcription factor NRF2 (NF-E2-related factor 2) plays a central role in the inducible cytoprotective response to the oxidative insults [3,4]. Under basal unstressed conditions, the NRF2 protein level is maintained at relatively low level, due to constitutive ubiquitination of NRF2 by KEAP1 (Kelch-like ECH-associated protein 1), an adaptor component of a CUL3 (Cullin 3)-based ubiquitin E3 ligase complex. KEAP1 targets NRF2 for proteasomal degradation [[5], [6], [7]]. Upon exposure to reactive oxygen species (ROS) or electrophiles, the ubiquitin conjugation activity of KEAP1 and actual ubiquitination of NRF2 is repressed, which leads to the stabilization and nuclear translocation/accumulation of NRF2 [8], followed by the upregulation of antioxidant gene expression [9,10].

A multitude of NRF2 inducers have been reported, most of which are electrophilic and readily react with cysteine thiol groups in KEAP1 [11,12]. KEAP1 is a cysteine-rich protein possessing 27 and 25 cysteine residues in the human and mouse proteins, respectively. Series of labeling and mass spectrometry studies have identified covalent modifications of specific cysteine residues of KEAP1 by electrophiles [11,[13], [14], [15], [16], [17], [18]]. Functional significance of these cysteine residues has been examined in various experimental systems [12,15,[19], [20], [21], [22]].

The significance of these cysteine residues has been elucidated by the studies of KEAP1 mutant mice. We have identified that Cys151/Cys273/Cys288 play a fundamental role in the sensing of NRF2-inducing electrophilic chemicals through C–S bond formation between cysteine residues and electrophile [11,23]. Based on the functional necessity of these three residues, electrophilic inducers of NRF2 are categorized into the three classes: Class I, Cys151-dependent compounds exemplified by sulforaphane (SFN), dimethyl-fumarate (DMF), and 1-[2-cyano-3,12-dioxooleana-1,9(11)-dien-28-oyl] imidazole (CDDO-Im). Class II includes 15-deoxy-Δ^12,14^-prostaglandin J_2_ (15d-PGJ_2_), which utilizes Cys288. Class III consists of 4-hydroxy-nonenal (4-HNE), sodium meta-arsenite (NaAsO_2_) and 9-nitro-octadec-9-enoic acid (9-OA-NO_2_), which react with any of the three sensor cysteines, Cys151/Cys273/Cys288 [23].

An intriguing observation here is that, apart from the electrophile-sensors, four cysteine residues of KEAP1, i.e., Cys226, Cys613, Cys622 and Cys624 are identified as sensors for hydrogen peroxide (H_2_O_2_) by the systematic mutagenesis experiment of KEAP1 using mouse embryonic fibroblasts (MEFs) [24]. These cysteine residues act distinctly from the electrophilic sensor cysteines, so that we have been proposing to classify reactive oxygen species (ROS) as Class IV of NRF2-inducing chemicals [8]. Analyses of the MEFs expressing a series of KEAP1 mutants revealed that KEAP1 utilizes these cysteine residues redundantly to realize a fail-safe mechanism that enables any combination of Cys226, Cys613, and Cys622/624 to form a disulfide bond (S–S bond) for sensing H_2_O_2_. Namely, KEAP1^C226S&C613S^ mutant can repress the basal NRF2 activity and activate the NRF2 pathway in response to electrophilic NRF2-inducing chemicals such as Class I inducers, but fails to activate the NRF2 pathway in response to Class IV inducer H_2_O_2_ [24]. Because oxidative stress appears to play important roles in cellular physiology as well as to play key roles in the development of multiple human diseases [1], physiological importance of the KEAP1 oxidative stress sensors needs to be investigated.

Despite these decent progresses in the analyses of KEAP1 sensor activities, characterizations of the KEAP1 sensor activities *in vivo* under physiological and pathological conditions remain to be clarified. Of the many questions left, we are especially keen to address following three questions, i) whether these KEAP1 sensor systems are operating *in vivo* as the main pathways, ii) whether oxidative and electrophilic stresses are sensed by using the similar system or sensed by distinct systems in a mutually non-overlapping manner, and iii) how KEAP1 equips sensitive and elaborate mechanisms that detect oxidative and electrophilic stresses *in vivo*.

In this study, therefore, we challenged a series of analyses utilizing conditional *selenocysteine-tRNA* (*Trsp*) knockout mice, KEAP1 sensor cysteine substitution mutant mice, and human cohort whole genome sequence (WGS) data to investigate the contributions of the KEAP1 oxidative stress sensor *in vivo*. The results unequivocally demonstrate following three points. Firstly, the KEAP1 oxidative stress sensor is essential for the compensatory activation of NRF2, maintenance of liver homeostasis, and overall survival of the hepatocyte-specific *Trsp*-deficient mice. Secondly, this study also provides lines of evidence for the differential utilization of KEAP1 cysteine sensors for oxidative and electrophiles stresses *in vivo*. Thirdly, this study revealed deep mechanistic insights in which oxidative modification of a single molecule of KEAP1 within the KEAP1 dimer is sufficient to inactivate KEAP1 ubiquitin ligase function, thereby NRF2 activation, implying that the KEAP1-NRF2 system serves as an extraordinary sensitive sensor for oxidative stress. Human genome analyses utilizing the Japanese multi-omics reference panel (jMorp), covering WGS information of Tohoku Medical Megabank (TMM) 54KJPN panel [25] and gnomAD Global panel [26] identified seven rare variants of the oxidative stress sensor residues in human KEAP1, further supporting the presence of fail-safe mechanism in the KEAP1 oxidative stress sensor activity. These results provide lines of evidence how mammals respond efficiently and separately to oxidative and electrophilic stresses.

## 2 Methods

### 2.1 Mice

All mice were treated according to the regulation of The Standards for Human Care and Use of Laboratory Animals of Tohoku University (Sendai, Japan) and the Guidelines for Proper Conduct of Animal Experiments of the Ministry of Education, Culture, Sports, Science, and Technology of Japan. All animal experiments were executed with the approval of the Tohoku University Animal Care Committee. *Alb*Cre mice [49], *Trsp*^*fl*^ mice [28], *Keap1*^*C226S&C613S*^ mice [24], *Keap1*^*C151S*^ mice [23], *Keap1*^*–*^ mice [50] and *Keap1*^*FA*^ mice [51] have been described. The genotyping was performed by PCR or TaqMan real-time quantitative PCR (qPCR) (Life Technologies). Sequence information of the genotyping primers and probes are shown in Supplementary Table 1.

### 2.2 CDDO-Im administration

CDDO-Im (HY-15725; MedChemExpress) was first dissolved into dimethyl sulfoxide (DMSO), and the DMSO containing CDDO-Im was mixed with Cremophor-EL and phosphate buffered saline (PBS) at the ratio of 1:1:8 (DMSO: Cremophor-EL: PBS) and the final concentration of CDDO-Im in this mixture was 3 mmol/L [52,53]. The CDDO-Im mixture was administered at a dose of 10 mL per kg BW orally to the male mice at 6–8 weeks of age (Fig. 4A–C) or 14 days of age (Fig. 4D). Livers were collected from each mouse 24 h after the administration.

### 2.3 Western blot analysis

Livers of male mice were homogenized in 9 vol of 0.25-M sucrose. For whole liver lysates, the homogenate was diluted with 2x sample buffer containing 20% glycerol, 4% SDS, 0.1-M Tris-HCl [pH 6.8], 12% 2-mercaptoethanol (2-ME), 0.88-mM phenylmethyl sulfonyl fluoride, cOmplete^TM^ EDTA-free protease inhibitor cocktail tablet (Roche) and bromophenol blue. For nuclear fractionation, the homogenate was centrifuged at 600×*g* for 10 min. The pellet (nuclear fraction) was suspended in the 2x sample buffer. For redox Western blot, livers were homogenized in 4 vol of redox lysis buffer containing 0.1-M Tris-HCl [pH 8.0], 0.12-M NaCl, 0.2% deoxycholic acid, 5% IGEPAL® CA-630, 0.2-mM sodium fluoride, 0.2-mM EDTA, 0.1-mM phenylmethyl sulfonyl fluoride, 40-mM N-ethylmaleimide and cOmpleteTM EDTA-free protease inhibitor cocktail tablet. The homogenate was diluted with 2 vol of 3x loading buffer containing 45% glycerol, 6% SDS, 0.2-M Tris-HCl [pH 6.8] and bromophenol blue. Half of the sample was reduced by the addition of 2-ME (6% v/v). After heat denaturation, the protein samples were subjected to SDS-polyacrylamide gel electrophoresis (SDS-PAGE) and electro-transferred to PVDF membranes. Specific protein signals were detected by anti-NQO1 (ab2346, Abcam; 1:3000 dilution), anti-GPX1 ([54]; 1:2000 dilution), anti-GPX4 (ab125066, Abcam; 1:1000 dilution), anti-KEAP1 ([55]; 1:200 dilution), anti-α-TUBULIN (T9026, Sigma-Aldrich; 1:1000 dilution), anti-NRF2 ([56]; 1:1000 dilution) or anti-LAMIN B1 (SC-374015, Santa Cruz; 1:1000 dilution) antibodies. Band intensities of KEAP1 were quantified using Image Lab^TM^ Software Version 5.0 (Bio-Rad).

### 2.4 Gene expression analysis

Total RNA was extracted from livers of male mice using Sepasol®-RNA I Super G (09379-97; Nacalai Tesque). The RNA concentration was measured using a NanoPhotometer® NP800 (Implen). RNA was reverse transcribed into cDNAs using ReverTra Ace® qPCR RT Master Mix with gDNA Remover (FSQ-301; Toyobo) according to the manufacturer’s instructions. The resulting cDNA was used as a template for reverse transcription qPCR (RT-qPCR) using a THUNDERBIRD® Probe qPCR Mix (QPS-101, Toyobo) with a QuantStudio® (Life Technologies). The *Hprt* gene was used as an internal control. Gene-specific primer and probe sequences are shown in Supplementary Table 2.

### 2.5 RNA-sequence analysis

Total RNA was extracted from livers using Sepasol®-RNA I Super G. RNA quantity and purity were assessed using NanoPhotometer® NP800 (Implen). RNA integrity (RIN) was assessed using TapeStation®(Agilent). rRNA fraction was removed using an MGIEasy rRNA depletion kit®(MGI). Double-stranded DNA (dsDNA) libraries were created from the rRNA-depleted eluate using an MGIEasy RNA directional library prep kit®(MGI). The libraries were sequenced on a DNBSEQ-G400RS®(MGI) system with a DNBSEQ-G400RS high-throughput sequencing kit V1.0 (MGI) to obtain 150 paired-end reads. The sequencing data was processed on the Galaxy server (https://usegalaxy.org) [57]. Transcript per million (TPM) normalized read counts were generated using Salmon quant script (Galaxy version 1.5.1+galaxy0) [58] with mouse reference transcript sequences (version 30) downloaded from GENCODE database (https://www.gencodegenes.org) [59]. The differential gene expression analysis was conducted on the iDEP platform (http://bioinformatics.sdstate.edu/idep/) [60]. RNA-seq data are available at GEO with an accession number GSE276818.

### 2.6 Biochemical analysis

Blood samples obtained from inferior vena cava were analyzed. Plasma alanine aminotransferase (ALT) values were determined using FUJI-DRI-CHEM 7000V (FUJIFILM).

### 2.7 Histological analysis

For hematoxylin-eosin (HE) staining, livers were fixed with Mildform® 10 N (131–10317; Wako Pure Chemical). The fixed tissues were embedded in paraffin, sliced into 4-μm-thick sections, and stained with HE. For immunohistochemistry, paraffin sections were rehydrated, autoclaved in 10-mmol/L sodium citrate buffer (pH 6.0) for antigen retrieval, treated with 3% H_2_O_2_, blocked with Protein Block Serum-free (X0909; Dako), and sequentially incubated with primary antibodies using anti-NQO1 (ab2346, Abcam; 1:500 dilution) for 16 h at 4 °C and the corresponding secondary antibodies.

### 2.8 jMorp database search

Genetic variants of human KEAP1 and their allele frequency from both TMM 54KJPN panel [25] and gnomAD [26] were searched in jMorp web database (https://jmorp.megabank.tohoku.ac.jp/).

### 2.9 Statistical analysis

Data were analyzed by one-way ANOVA followed by Tukey-Kramer HSD test using JMP® Pro 17.1.0. P < 0.05 was considered statistically significant; *∗P* < 0.05, *∗∗P* < 0.01, *∗∗∗P* < 0.0001.

## 3 Results

**KEAP1-Cys226/613 is required for increased NQO1 expression in selenoprotein deficient liver**. Selenoproteins such as glutathione peroxidase (GPX) and thioredoxin reductase (TXNRD) are important for maintaining intracellular redox balance. Selenocysteine tRNA (tRNA^SeCys^) is essential for the selenoprotein synthesis [27]. Deletion of the *tRNA*^*SeCys*^ (*Trsp*) gene causes a failure in the selenoprotein synthesis, resulting in increased intracellular ROS, such as H_2_O_2_ [28,29], and S–S bond formation of proteins, since the TXNRD-dependent thioredoxin system is a main disulfide reducing system [30]. The NRF2 pathway is activated in the hepatocyte-specific selenoprotein deficiency brought by the *Trsp* gene deletion [28]. However, how selenoprotein deficiency triggers the NRF2-mediated compensation remains unknown. Considering that S–S bond formation between KEAP1 oxidative stress sensor cysteines is important for oxidative stress sensing, we hypothesized that selenoprotein deficiency activates NRF2 through formation of S–S bond between the oxidative stress sensor cysteine residues in KEAP1.

To test this hypothesis, we decided to utilize KEAP1^C226S&C613S^ mutant mice, in which both Cys226 and Cys613 residues are replaced with serine residues [24]. Analyses of the MEFs from *Keap1*^*C226S&C613S/C226S&C613S*^ mice, hereafter referred to as *Keap1*^*C226S&C613S homo*^ mice, have revealed that KEAP1-C226S&C613S mutant protein was able to normally repress NRF2 but failed to activate NRF2 in response to H_2_O_2_ [24]. In addition, H_2_O_2_ treatment has been shown to induce formation of S–S bond between Cys226 and Cys613 in KEAP1 [31]. Therefore, to examine the contribution of KEAP1-Cys226/613 to the NRF2 activation in selenoprotein deficient livers, we generated compound mutant mice by crossing conditional liver-specific *Trsp* knockout (*Trsp*^*fl/fl*^::*Alb*Cre) mice and KEAP1^C226S&C613S^ mutant mice.

**Fig. 1:**
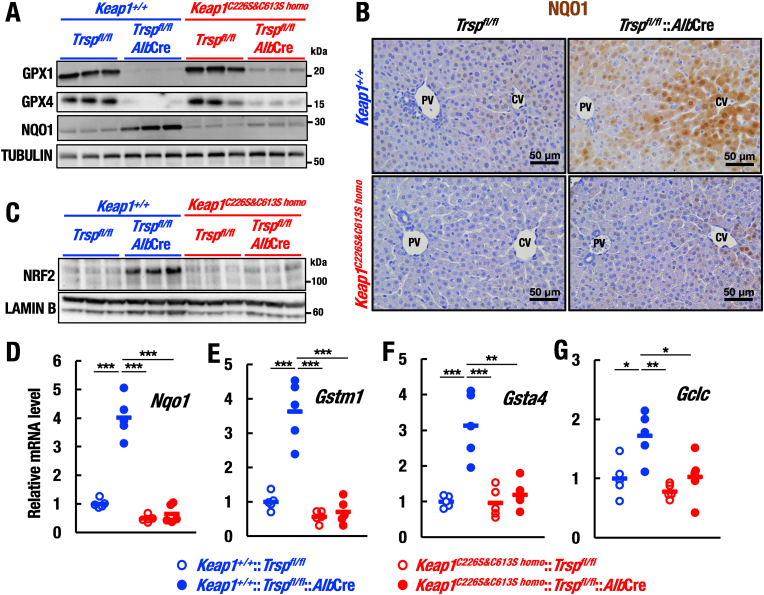
KEAP1-Cys226/613 is required for NRF2 activation in selenoprotein-deficient mouse livers. (A) Protein levels of selenoprotein members GPX1 and GPX4, and non-selenium containing enzyme NQO1 in whole liver lysates of *Trsp*^*fl/fl*^, *Trsp*^*fl/fl*^::*Alb*Cre, *Keap1*^*C226S&C613S homo*^::*Trsp*^*fl/fl*^ and *Keap1*^*C226S&C613S homo*^::*Trsp*^*fl/fl*^:*Alb*Cre mice at 20 days of age were analyzed by Western blot. (B) Representative images of NQO1 immunostaining of livers from *Trsp^fl/fl^*, *Trsp^fl/fl^*::*Alb*Cre, *Keap1*^*C226S&C613S*^^*homo*^::*Trsp*^*fl/fl*^ and *Keap1*^*C226S&C613S homo*^::*Trsp*^*fl/fl*^:*Alb*Cre mice at 20 days of age. CV and PV indicate central veins and portal veins, respectively. (C) Nuclear NRF2 protein levels in livers of *Trsp*^*fl/fl*^, *Trsp*^*fl/fl*^::*Alb*Cre, *Keap1*^*C226S&C613S homo*^::*Trsp*^*fl/fl*^ and *Keap1*^*C226S&C613S homo*^::*Trsp*^*fl/fl*^::*Alb*Cre mice at 20 days of age were analyzed by Western blot. (D–G) Relative mRNA levels of *Nqo1* (D), *Gstm1* (E), *Gsta4* (F) and *Gclc* (G) in livers of *Trsp*^*fl/fl*^, *Trsp*^*fl/fl*^::*Alb*Cre, *Keap1*^*C226S&C613S homo*^::*Trsp*^*fl/fl*^ and *Keap1*^*C226S&C613S homo*^::*Trsp*^*fl/fl*^::*Alb*Cre mice (n = 5, each) at 20 days of age were analyzed by RT-qPCR. The data were analyzed by one-way ANOVA followed by Tukey-Kramer HSD test (*∗P* < 0.05, *∗∗P* < 0.01 and *∗∗∗P* < 0.0001).

To validate the disruption of selenoprotein synthesis in the compound mutant mice, we first examined GPX1 and GPX4 protein levels, and found that the expressions of GPX1 and GPX4 proteins were markedly decreased in *Trsp*^*fl/fl*^::*Alb*Cre mouse livers compared to control *Trsp*^*fl/fl*^ mouse livers (Fig. 1A). The expressions of GPX1 and GPX4 were also decreased in *Keap1*^*C226S&C613S homo*^::*Trsp*^*fl/fl*^::*Alb*Cre mouse livers, indicating that deletion of the *Trsp* gene causes disruption in the synthesis of selenium-containing antioxidant enzymes. Importantly, we found that protein level of NAD(P)H:quinone oxidoreductase 1 (NQO1), a representative NRF2 target gene, was significantly increased in *Trsp*^*fl/fl*^::*Alb*Cre mouse liver (Fig. 1A), consistent with our previous study [28]. By contrast, the elevation of NQO1 protein level was markedly diminished in *Keap1*^*C226S&C613S homo*^::*Trsp*^*fl/fl*^::*Alb*Cre mouse livers (Fig. 1A), supporting the previous finding that KEAP1-Cys226/613 acts as a sensor for H_2_O_2_ [24].

Immunohistochemical analysis also revealed that NQO1 was strongly induced in hepatocytes in the proximity of central vein (CV) compared to that in the portal vein (PV) regions in *Trsp*^*fl/fl*^::*Alb*Cre mouse livers (Fig. 1B). This observation is in good agreement with the observation that NRF2 activity is higher in hepatocytes surrounding the CV than in those surrounding the PV [32]. By contrast, the induced NQO1 protein level was weakened in *Keap1*^*C226S&C613S homo*^::*Trsp*^*fl/fl*^::*Alb*Cre mouse livers (Fig. 1B). These results demonstrate that KEAP1-Cys226/613 is required for the elevation of NQO1 protein in response to selenoprotein deficiency in liver.

**KEAP1-Cys226/613 is required for NRF2 activation in selenoprotein deficient liver**. The result that NQO1 protein upregulation depends on the presence of KEAP1-Cys226/613 in selenoprotein deficiency suggests that NRF2 protein level is regulated by the signal sensed and transduced by the KEAP1-Cys226/613. To further verify this hypothesis, we examined nuclear NRF2 protein level in mice (Fig. 1C). Consistent with the previous study [28], NRF2 protein level was increased in *Trsp*^*fl/fl*^::*Alb*Cre mouse livers. Importantly, however, the NRF2 accumulation was significantly diminished in *Keap1*^*C226S&C613S homo*^::*Trsp*^*fl/fl*^::*Alb*Cre mouse livers (Fig. 1C), indicating that the KEAP1-Cys226/613 is essential for the NRF2 activation provoked by the selenoprotein deficiency in liver.

To ascertain the contribution of KEAP1-Cys226/613 to the NRF2 activation by the selenoprotein deficiency, we examined transcript levels of the representative NRF2 target genes, including *Nqo1*, *Glutathione S-transferase mu 1* (*Gstm1*), *Glutathione S-transferase alpha 4* (*Gsta4*) and *Glutamate-cysteine ligase catalytic subunit* (*Gclc*) (Fig. 1D–G). Results showed that expressions of NRF2 target genes were increased in *Trsp*^*fl/fl*^::*Alb*Cre mice compared to those in control *Trsp*^*fl/fl*^ mice (Fig. 1B–E). In contrast, inductions of these NRF2 target genes in *Trsp*^*fl/fl*^::*Alb*Cre mouse livers were markedly reduced in *Keap1*^*C226S&C613S homo*^::*Trsp*^*fl/fl*^::*Alb*Cre mouse livers. These results demonstrates that the KEAP1-Cys226/613 is critical for induction of NRF2 activity under selenoprotein deficiency.

**Fig. 2:**
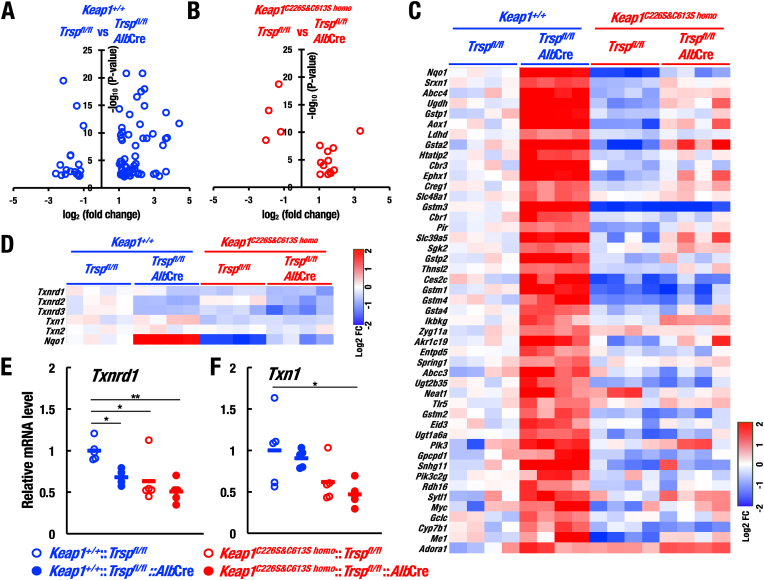
KEAP1-Cys226/613 predominantly contributes to the cytoprotective gene expression under selenoprotein-deficiency. (A) Volcano plots of differentially expressed genes (absolute log2 fold-change >1 and adjusted p-value <0.01) in livers of *Trsp*^*fl/fl*^::*Alb*Cre versus *Trsp*^*fl/fl*^ mice at 20 days of age were analyzed by using RNA-sequence data. (B) Regarding genes that were differentially expressed in livers of *Trsp*^*fl/fl*^::*Alb*Cre versus *Trsp*^*fl/fl*^ mice in (A), volcano plots of differentially expressed genes (absolute log2 fold-change >1 and adjusted p-value <0.01) in livers of *Keap1*^*C226S&C613S homo*^::*Trsp*^*fl/fl*^::*Alb*Cre versus *Keap1*^*C226S&C613S homo*^::*Trsp*^*fl/fl*^ mice. (C) Heatmap of the genes up-regulated in *Trsp*^*fl/fl*^::*Alb*Cre mouse livers compared with those of *Trsp*^*fl/fl*^ mouse livers after qualification of the genes extracted from volcano plot analysis. Note that most of the gene expressions were dependent on KEAP1-Cys226/613. (D) Heatmap of *Txnrd* and *Txn* genes drawn by using RNA-sequence data. Their mRNA levels are not induced by selenoprotein deficiency. (E–F) Relative mRNA levels of *Txnrd1* (E) and *Txn1* (F) in livers of *Trsp*^*fl/fl*^, *Trsp*^*fl/fl*^::*Alb*Cre, *Keap1*^*C226S&C613S homo*^::*Trsp*^*fl/fl*^ and *Keap1*^*C226S&C613S homo*^::*Trsp*^*fl/fl*^::*Alb*Cre mice (n = 5, each) at 20 days of age were analyzed by RT-qPCR. The data were analyzed by one-way ANOVA followed by Tukey-Kramer HSD test (*∗P* < 0.05 and *∗∗P* < 0.01).

**KEAP1 acts as the main sensor of selenoprotein deficiency**. To examine contribution of KEAP1-Cys226/613 to the gene expression in selenoprotein-deficient livers, we conducted RNA-sequencing analysis. Volcano plot analysis of the results showed that 58 up-regulated genes and 19 down-regulated genes in *Trsp*^*fl/fl*^::*Alb*Cre mouse livers compared with control *Trsp*^*fl/fl*^ mouse livers (absolute log2 fold-change >1 and adjusted p-value <0.01; Fig. 2A). By contrast, among the differentially changed genes between *Trsp*^*fl/fl*^::*Alb*Cre and *Trsp*^*fl/fl*^ mouse livers, the substitutive mutations of KEAP1-Cys226/613 elicited marked decrease in the numbers of differential expression genes, only 12 up-regulated genes and 4 down-regulated genes, in *Keap1*^*C226S&C613S homo*^::*Trsp*^*fl/fl*^::*Alb*Cre mouse livers compared with control *Keap1*^*C226S&C613S homo*^::*Trsp*^*fl/fl*^ mouse livers (Fig. 2B). These results further support the notion that KEAP1-Cys226/613 contributes to major part of the gene expression regulation elicited by the selenoprotein deficiency. Down-regulated genes in both *Trsp*^*fl/fl*^::*Alb*Cre and *Keap1*^*C226S&C613S homo*^::*Trsp*^*fl/fl*^::*Alb*Cre mouse livers include selenoprotein genes, such as *Gpx1*, *Selenow* and *Selenop* (Fig. S1), confirming that impaired synthesis of selenoprotein in fact leads to non-sense mediated decay of selenoprotein mRNAs [33].

To examine differentially expressed genes elicited by the selenoprotein deficiency, we generated a heatmap of the up-regulated genes in *Trsp*^*fl/fl*^::*Alb*Cre mouse livers (Fig. 2C). Many canonical and well-known NRF2 target genes, including *Malic enzyme 1* (*Me1*), *Sulfiredoxin 1* (*Srxn1*), *Nqo1* and *Gclc* [10], reside in the up-regulated genes in *Trsp*^*fl/fl*^::*Alb*Cre mouse livers. These genes coordinately contribute to maintain intracellular redox balance, as ME1, SRXN1 and GCLC are involved in NADPH production, oxidoreductase activity, and glutathione synthesis. Of note, the heatmap indicates that most of the up-regulations of the genes in *Trsp*^*fl/fl*^::*Alb*Cre mouse livers were cancelled in *Keap1*^*C226S&C613S homo*^::*Trsp*^*fl/fl*^::*Alb*Cre mouse livers.

**Fig. 3:**
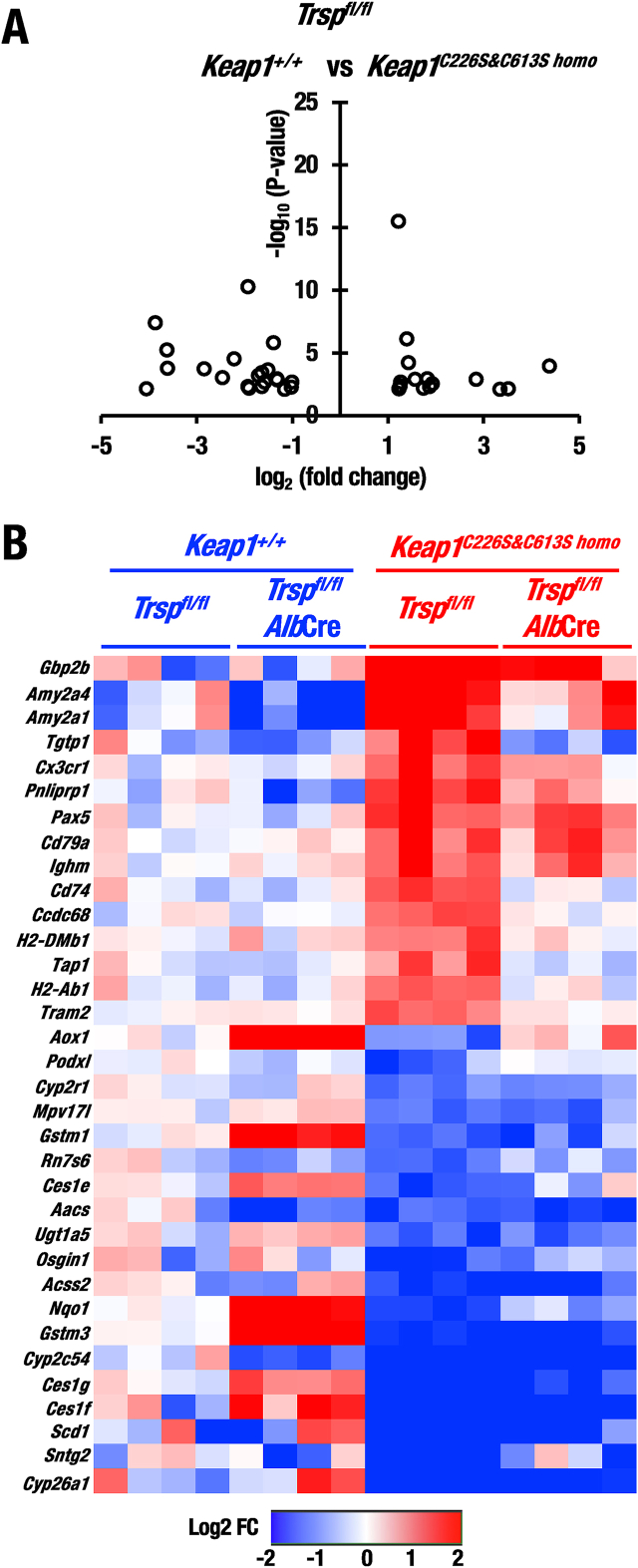
The differentially expressed genes in livers of *Keap1*^*C226S&C613S homo*^::*Trsp*^*fl/fl*^ versus *Keap1*^*+/+*^::*Trsp*^*fl/fl*^ mice. (A) Volcano plot of differentially expressed genes (absolute log2 fold-change >1 and adjusted p-value <0.01) in livers of *Keap1*^*C226S&C613S homo*^::*Trsp*^*fl/fl*^ versus *Keap1*^*+/+*^::*Trsp*^*fl/fl*^ mice at 20 days of age were analyzed by using RNA-sequence data. This result shows that 15 up-regulated genes and 23 down-regulated genes in *Keap1*^*C226S&C613S homo*^::*Trsp*^*fl/fl*^ mouse livers compared with control *Keap1*^*+/+*^::*Trsp*^*fl/fl*^ mouse livers. (B) Heatmap of the genes up-regulated and down-regulated in *Keap1*^*C226S&C613S homo*^::*Trsp*^*fl/fl*^ mouse livers compared with those of *Keap1*^*+/+*^::*Trsp*^*fl/fl*^ mouse livers after qualification of the genes extracted from volcano plot analysis.

An intriguing observation here is that *Txnrd1* and *Txn1*, which are also known NRF2 target genes, were not induced in *Trsp*^*fl/fl*^::*Alb*Cre mouse livers (Fig. 2D–F). This observation implies that some other systems may contribute to the NRF2-mediated compensation of redox balance in the selenoprotein deficient livers. Another interesting observation is that the expressions of NRF2-target genes were decreased in *Keap1*^*C226S&C613S homo*^::*Trsp*^*fl/fl*^ mouse livers compared to control *Trsp*^*fl/fl*^ mouse livers (Fig. 2C), indicating that lack of KEAP1-Cys226/613 fails to sense basal level of oxidative stresses. Supporting this finding, volcano plot analysis showed that 23 down-regulated genes in *Keap1*^*C226S&C613S homo*^::*Trsp*^*fl/fl*^ mouse livers compared with control *Trsp*^*fl/fl*^ mouse livers (Fig. 3A). The down-regulated genes include canonical NRF2-target genes such as *Nqo1* and *Gstm1* (Fig. 3B). These results suggest that the KEAP1-Cys226/613 contributes to the maintenance of basal or steady-state level NRF2 activity in addition to the response to oxidative stress under selenoprotein-deficient conditions.

**Fig. 4:**
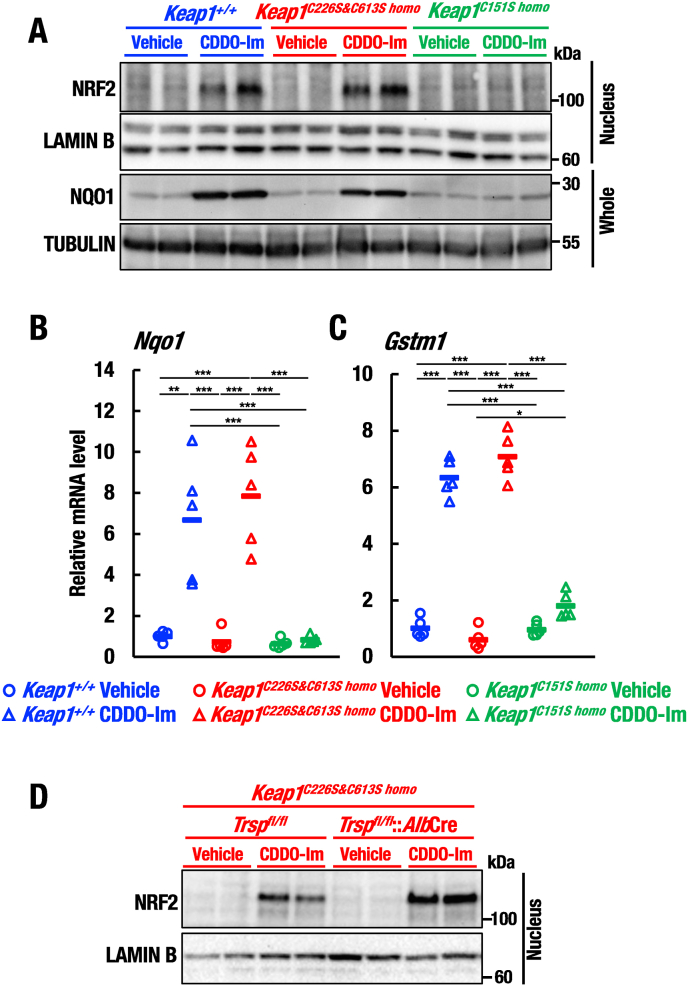
KEAP1-Cys226/613 is dispensable for sensing CDDO-Im *in vivo*. (A) Protein levels of NRF2 and NQO1 in livers of *Keap1*^*+/+*^, *Keap1*^*C226S&C613S homo*^ and *Keap1*^*C151S homo*^ mice orally treated with 30 μmol/kg/body weight CDDO-Im or vehicle at 6–8 weeks of age were analyzed by Western blot. (B, C) Relative mRNA levels of *Nqo1* (B) and *Gstm1* (C) in livers of *Keap1*^*+/+*^, *Keap1*^*C226S&C613S homo*^ and *Keap1*^*C151S homo*^ mice orally treated with 30 μmol/kg/body weight CDDO-Im or vehicle at 6–8 weeks of age were analyzed by RT-qPCR. n = 5 each group. The data were analyzed by one-way ANOVA followed by Tukey-Kramer HSD test (*∗P* < 0.05, *∗∗P* < 0.01 and *∗∗∗P* < 0.0001). (D) Nuclear NRF2 protein levels in livers of *Keap1*^*C226S&C613S homo*^::*Trsp*^*fl/fl*^ and *Keap1*^*C226S&C613S homo*^::*Trsp*^*fl/fl*^::*Alb*Cre mice orally treated with 30 μmol/kg/body weight CDDO-Im or vehicle at 14 days of age were analyzed by Western blot. Note that CDDO-Im treatment accumulates NRF2 independently of KEAP1-Cys226/613 and selenoproteins.

**KEAP1-Cys226/613 is dispensable for electrophile-mediated NRF2 activation**. One important remaining question is whether the KEAP1-Cys226/613 acts exclusively for the oxidative stress sensing, or it has cross reactivity to certain extent for the electrophilic stress sensing. It has been shown that KEAP1-Cys151 is the important sensor for the Class I group of electrophiles, including CDDO-Im [23,34,35], which is structural analogue of the recently approved Omaveloxolone for the treatment of Friedreich’s ataxia patients [36]. Therefore, to investigate whether KEAP1-Cys226/613 contributes to the sensing of KEAP1-Cys151-preferring electrophile CDDO-Im or not, we examined CDDO-Im-mediated NRF2 activation *in vivo* by using KEAP1-Cys226/613 substitution mutant mice.

We found that oral administration of CDDO-Im induced accumulation of nuclear NRF2 protein in *Keap1*^*C226S&C613S homo*^ mouse livers in a similar extent to the wild-type mouse livers (Fig. 4A), indicating that KEAP1-Cys226/613 is dispensable for the CDDO-Im-mediated NRF2 activation. By contrast, CDDO-Im induced the NRF2 accumulation markedly less in *Keap1*^*C151S homo*^ mouse livers than in *Keap1*^*C226S&C613S homo*^ and wild-type mouse livers (Fig. 4A). These changes were nicely reproducible in the analysis of NQO1 expression (Fig. 4A). These results clearly demonstrate that KEAP1-Cys226/613 is dispensable, but KEAP1-Cys151 is indispensable for the CDDO-Im-mediated induction of NRF2.

To further validate induction of transcriptional activity of NRF2, we examined transcript levels of NRF2 target genes in livers of *Keap1*^*C226S&C613S homo*^ and *Keap1*^*C151S homo*^ mice treated with CDDO-Im (Fig. 4B and C). Showing very good agreement with the Western blot analysis, expression levels of *Nqo1* and *Gstm1* in *Keap1*^*C226S&C613S homo*^ mice treated with CDDO-Im were comparable with those of wild-type mice treated with CDDO-Im (Fig. 4B and C). By contrast, expression levels of *Nqo1* and *Gstm1* in CDDO-Im-treated *Keap1*^*C151S homo*^ mice were significantly diminished compared with those of wild-type mice treated with CDDO-Im (Fig. 4B and C). Thus, these results provide lines of evidence that KEAP1 utilizes the sensor cysteine residues differentially for detection of the oxidative and electrophilic stresses.

**CDDO-Im treatment accumulates NRF2 independently of KEAP1-Cys226/613 and selenoproteins**. CDDO-Im-mediated NRF2 activation is dependent on KEAP1-Cys151, but independent of KEAP1-Cys226/613, whereas KEAP1-Cys226/613 is critical for the compensatory activation of NRF2 against the selenoprotein deficiency. To address whether the CDDO-Im treatment can rescue the defect of NRF2 activation in livers of the KEAP1^C226S&C613S^ mutant mice with *Trsp* deficiency, we examined livers of *Keap1*^*C226S&C613S homo*^::*Trsp*^*fl/fl*^::*Alb*Cre mice treated with CDDO-Im. Because *Keap1*^*C226S&C613S homo*^::*Trsp*^*fl/fl*^::*Alb*Cre mice did not survive long after birth, we decided to use mice at 14 days of age, when more than half of the mice were alive, and treated the mice with CDDO-Im.

Oral administration of CDDO-Im to *Keap1*^*C226S&C613S homo*^::*Trsp*^*fl/fl*^ mice induced NRF2 protein level compared to the vehicle-treated mice (Fig. 4D), indicating that KEAP1-Cys226/613 is dispensable for the NRF2 activation mediated by CDDO-Im. Of note, the CDDO-Im treatment increased the NRF2 protein level in the *Keap1*^*C226S&C613S homo*^::*Trsp*^*fl/fl*^::*Alb*Cre mouse livers compared to the vehicle-treated control mice (Fig. 4D). These results demonstrate that CDDO-Im acts to accumulate NRF2 via intact KEAP1-Cys151 even in the absence of KEAP1-Cys226/613, and strongly support the notion that KEAP1 differentially utilizes the sensor cysteine residues for detection of oxidative and electrophilic stresses, but the selenoproteins are dispensable for the CDDO-Im-mediated NRF2 accumulation.

**Fig. 5:**
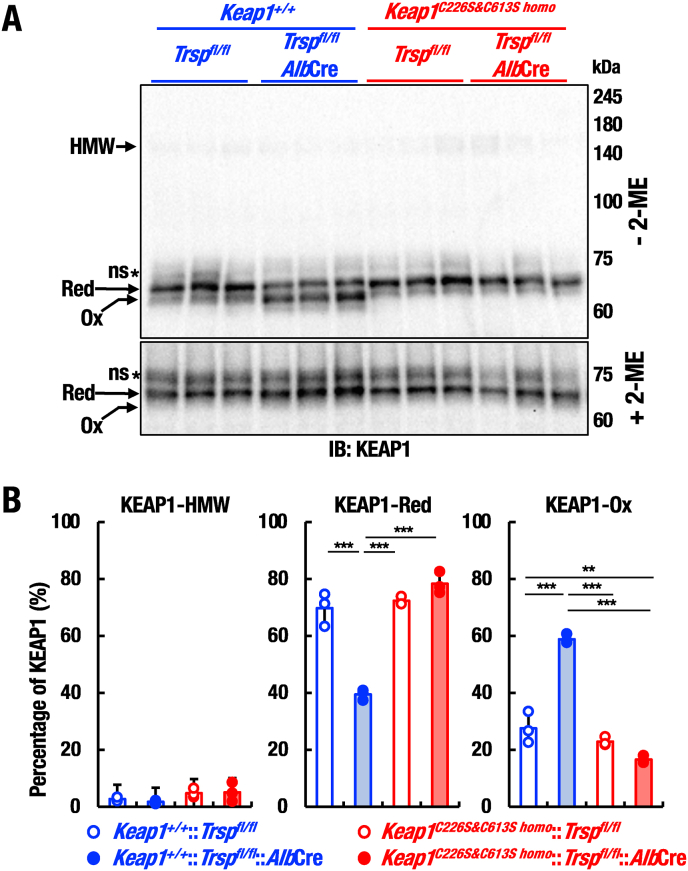
KEAP1-Cys226/613 is oxidized in selenoprotein-deficient livers. (A) Redox states of KEAP1 in livers of *Trsp*^*fl/fl*^, *Trsp*^*fl/fl*^::*Alb*Cre, *Keap1*^*C226S&C613S homo*^::*Trsp*^*fl/fl*^ and *Keap1*^*C226S&C613S homo*^::*Trsp*^*fl/fl*^::*Alb*Cre mice at 20 days of age were analyzed by Western blot with or without 2-mercaptoethanol (2-ME). HMW indicates high-molecular weight form of KEAP1 (approximately 150 kDa), which is corresponding to the KEAP1-Cys151-mediated intermolecular disulfide-binding form. Red and Ox in the left represent reduced and oxidized forms of KEAP1, respectively. Ox band shows faster migration than Red band or reduced form of KEAP1, which is corresponding to the KEAP1-Cys226/613-mediated intramolecular disulfide-bond formation. ns stands for a non-specific band. (B) Graphical presentation of HMW, Red and Ox form of KEAP1 percentages shown in (A). Note that approximately 50% of KEAP1 is oxidized (i.e., Ox KEAP1) in *Trsp*^*fl/fl*^::*Alb*Cre mice, but the Ox form percentage significantly reduced by the lack of KEAP1-Cys226/613 in *Keap1*^*C226S&C613S homo*^::*Trsp*^*fl/fl*^ and *Keap1*^*C226S&C613S homo*^::*Trsp*^*fl/fl*^::*Alb*Cre mice. The data were analyzed by one-way ANOVA followed by Tukey-Kramer HSD test (*∗∗P* < 0.01 and *∗∗∗P* < 0.0001).

**KEAP1-Cys226/613 is oxidized in selenoprotein-deficient liver**. It has been reported that H_2_O_2_ treatment of cultured cells induces formation of intramolecular disulfide bond via KEAP1-Cys226/613 [31,37]. To clarify redox state of KEAP1 in selenoprotein-deficient livers *in vivo*, in this study we examined redox state of KEAP1 in livers by redox Western blot without adding reducing agent 2-mercaptoethanole (2-ME). We found that fast migrating band corresponding to the oxidized KEAP1 was significantly increased in *Trsp*^*fl/fl*^::*Alb*Cre mouse livers (Fig. 5A top panel, Ox). The oxidized form of KEAP1 disappeared by the addition of reducing agent, indicating that the oxidized form harbors reversible disulfide bond (Fig. 5A bottom panel).

Quantification of the band intensity demonstrated that the oxidized form of KEAP1 was significantly increased by the selenoprotein deficiency in a KEAP1-Cys226/613-dependent manner (Fig. 5B). The levels of oxidized form KEAP1 were significantly decreased in *Keap1*^*C226S&C613S homo*^::*Trsp*^*fl/fl*^::*Alb*Cre mouse livers compared with that in *Trsp*^*fl/fl*^::*Alb*Cre mouse livers (Fig. 5B). In addition, the levels of oxidized form KEAP1 in control *Trsp*^*fl/fl*^ mouse livers tend to be higher than those of *Keap1*^*C226S&C613S homo*^::*Trsp*^*fl/fl*^ mouse livers (Fig. 5A), implying that KEAP1 is partly but constitutively oxidized even at basal conditions and contributing to the maintenance of redox homeostasis. These results demonstrate that KEAP1-Cys226/613 is involved in the formation of oxidized KEAP1 in the untreated basal level livers as well as selenoprotein-deficient livers.

An interesting observation has been reported that, upon treatment with H_2_O_2_, intermolecular disulfide bonds were also formed in cultured cells via KEAP1-Cys151 [31]. To examine whether this is reproducible *in vivo*, we examined appearance of high molecular weight bands in *Trsp*^*fl/fl*^::*Alb*Cre mouse livers (Fig. 5A top panel, HMW). We could not find substantial increase of the HMW bands corresponding to the intermolecular disulfide bond formation in the selenoprotein-deficient livers. These results indicate that oxidation of KEAP1-Cys226/613 mainly contributes, but the intermolecular disulfide bond via KEAP1-Cys151 has rather minor contribution to the oxidative stress response in selenoprotein-deficient livers *in vivo*.

**Fig. 6:**
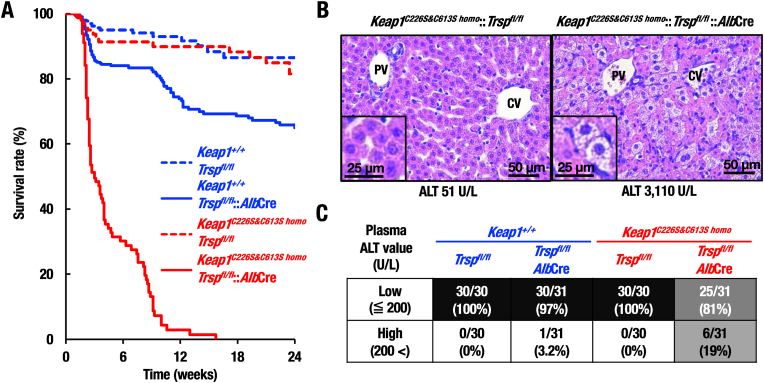
KEAP1-Cys226/613 is required for survival of hepatocyte-specific selenoprotein-deficient mice. (A) Survivals of *Trsp*^*fl/fl*^ (n = 395), *Trsp*^*fl/fl*^::*Alb*Cre (n = 424), *Keap1*^*C226S&C613S homo*^::*Trsp*^*fl/fl*^ (n = 164) and *Keap1*^*C226S&C613S homo*^::*Trsp*^*fl/fl*^::*Alb*Cre (n = 147) cohort of mice were monitored over a 24-week after birth period. (B) Representative images of HE staining of liver sections of *Keap1*^*C226S&C613S homo*^::*Trsp*^*fl/fl*^ and *Keap1*^*C226S&C613S homo*^::*Trsp*^*fl/fl*^::*Alb*Cre mice at 20 days of age. Insert images in *Keap1*^*C226S&C613S homo*^::*Trsp*^*fl/fl*^ and *Keap1*^*C226S&C613S homo*^::*Trsp*^*fl/fl*^::*Alb*Cre mouse liver sections indicate the presence of normal and ballooning hepatocytes, respectively. (C) Plasma ALT levels of *Trsp*^*fl/fl*^, *Trsp*^*fl/fl*^::*Alb*Cre, *Keap1*^*C226S&C613S homo*^::*Trsp*^*fl/fl*^ and *Keap1*^*C226S&C613S homo*^::*Trsp*^*fl/fl*^::*Alb*Cre mice at 12–24 days after birth were measured. Note the increase of mice with high ALT level in *Keap1*^*C226S&C613S homo*^::*Trsp*^*fl/fl*^::*Alb*Cre group, but the percentage of the mice is approximately 20%, indicating that the crisis of liver function occurs abruptly in these mice.

**KEAP1-Cys226/613 is essential for survival of hepatocyte-specific selenoprotein-deficient mice**. We have shown that *Nrf2*^*−/−*^::*Trsp*^*fl/fl*^::*Alb*Cre mice display poor survival [28]. In this study, we assessed whether the loss of NRF2 activation caused by the absence of KEAP1-Cys226/613 leads to poor survival of hepatocyte-specific selenoprotein-deficient mice. Kaplan-Meier curve indicates that most of the control *Keap1*^*C226S&C613S homo*^::*Trsp*^*fl/fl*^ mice survived 24 weeks after birth (Fig. 6A, red dot line). Similarly, approximately 70–80% of *Trsp*^*fl/fl*^::*Alb*Cre mice survived 24 weeks after birth (blue line), conforming our previous observation [28]. By contrast, most of the *Keap1*^*C226S&C613S homo*^::*Trsp*^*fl/fl*^::*Alb*Cre mice died within 10 weeks after birth (red line). The *Keap1*^*C226S&C613S homo*^::*Trsp*^*fl/fl*^::*Alb*Cre mice showed a clearly worse survival rate than *Trsp*^*fl/fl*^::*Alb*Cre mice did. These results demonstrate that the NRF2 activation mediated by the KEAP1-Cys226/613 critically contributes to the survival of animals in the selenoprotein deficient mouse livers.

**KEAP1-Cys226/613 is essential for maintenance of redox homeostasis of hepatocytes**. To examine hepatic damage in mice, we performed histological analyses of the livers. While control *Keap1*^*C226S&C613S homo*^::*Trsp*^*fl/fl*^ mice with low plasma ALT level displayed normal liver histology (Fig. 6B, left panel), *Keap1*^*C226S&C613S homo*^::*Trsp*^*fl/fl*^::*Alb*Cre mice with high plasma ALT level showed marked death of hepatocytes (Fig. 6B, right panel). The death of hepatocytes in *Keap1*^*C226S&C613S homo*^::*Trsp*^*fl/fl*^::*Alb*Cre mice was observed widely without biased accumulation in portal vein (PV) or central vein (CV) region. These results demonstrate that the KEAP1-Cys226/613 sensor function and resulting induction of NRF2 activity are essential for the maintenance of hepatocyte homeostasis under the selenoprotein deficiency.

In this survival study, we found that only 3.2% *Trsp*^*fl/fl*^::*Alb*Cre mice looked sick with high plasma ATL levels (> 200 U/L), but 97% *Trsp*^*fl/fl*^::*Alb*Cre mice displayed normal or low plasma ALT levels (≤ 200 U/L) at 12–24 days after birth (Fig. 6C). This observation shows very good coincidence with the observation that *Trsp*^*fl/fl*^::*Alb*Cre mice display normal plasma ALT level until approximately 24 h before their sudden death [38].

While loss of the KEAP1-Cys226/613 from *Trsp*^*fl/fl*^::*Alb*Cre mice markedly shortened the survival period of mice and most of the mice died before 12 weeks (Fig. 6A), only 19% *Keap1*^*C226S&C613S homo*^::*Trsp*^*fl/fl*^::*Alb*Cre mice appeared sick and displayed high plasma ALT levels in 12- to 24-day period after birth (Fig. 6C). An intriguing observation here is that remaining 81% of *Keap1*^*C226S&C613S homo*^::*Trsp*^*fl/fl*^::*Alb*Cre mice in this period appeared normal and displayed low plasma ALT levels, like the control *Trsp*^*fl/fl*^ mice and *Keap1*^*C226S&C613S homo*^::*Trsp*^*fl/fl*^ mice without Cre recombination. Thus, the death of *Keap1*^*C226S&C613S homo*^::*Trsp*^*fl/fl*^::*Alb*Cre mice occurred suddenly as was the cases for the death of *Trsp*^*fl/fl*^::*Alb*Cre mice, but the former cases occurred with much higher frequency. We surmise that the livers of the *Keap1*^*C226S&C613S homo*^::*Trsp*^*fl/fl*^::*Alb*Cre mice are usually in relatively unstressed conditions under threshold level of oxidative stress damage, which is presumably sustained by yet unidentified NRF2-independent and selenoprotein-independent antioxidant system. However, when certain stimuli trigger oxidative stress to deteriorate the liver function, the mice without the defense by selenoproteins and the KEAP1-NRF2 system cannot cope with the damage, leading to the abrupt death. This observation further demonstrates importance of the inducible nature of the KEAP1-NRF2 system even in the selenoprotein-deficient conditions.

**Fig. 7:**
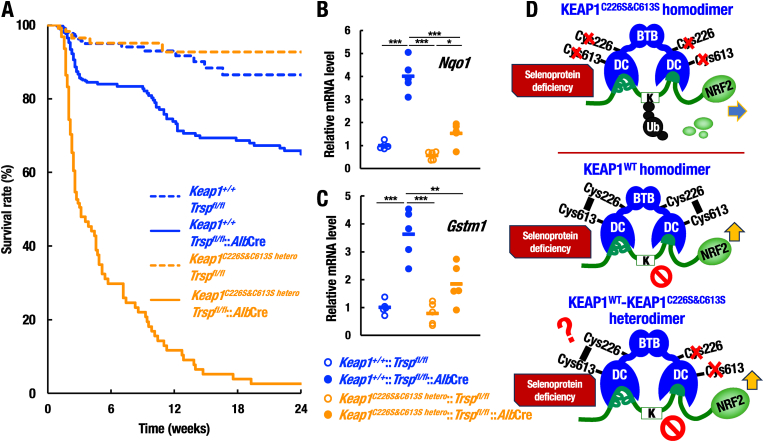
Heterozygous loss of KEAP1-Cys226/613 affects the NRF2-mediated compensation in selenoprotein-deficient liver. (A) Survivals of *Keap1*^*C226S&C613S hetero*^::*Trsp*^*fl/fl*^ (n = 114) and *Keap1*^*C226S&C613S hetero*^::*Trsp*^*fl/fl*^::*Alb*Cre (n = 125) cohort mice were monitored over a 24-week after birth period and the results were compared with those of *Trsp*^*fl/fl*^ (n = 395) and *Trsp*^*fl/fl*^::*Alb*Cre (n = 424) shown in Fig. 5A. (B, C) Relative mRNA levels of *Nqo1* (B) and *Gstm1* (C) in livers of *Keap1*^*C226S&C613S hetero*^::*Trsp*^*fl/fl*^ and *Keap1*^*C226S&C613S hetero*^::*Trsp*^*fl/fl*^::*Alb*Cre mice (n = 5, each) at 20 days of age were analyzed by RT-qPCR and compared with those of *Trsp*^*fl/fl*^ and *Trsp*^*fl/fl*^::*Alb*Cre shown in Fig. 1D and E. Note that the inductions of *Nqo1* and *Gstm1* expressions in *Keap1*^*C226S&C613S hetero*^::*Trsp*^*fl/fl*^::*Alb*Cre mouse livers were weaker than those of *Trsp*^*fl/fl*^::*Alb*Cre mouse livers, but mRNA levels of these genes in the *Keap1*^*C226S&C613S hetero*^::*Trsp*^*fl/fl*^::*Alb*Cre mouse livers were substantially higher than those of control *Keap1*^*C226S&C613S hetero*^::*Trsp*^*fl/fl*^ mouse livers, indicating that NRF2 is induced in *Keap1*^*C226S&C613S hetero*^::*Trsp*^*fl/fl*^::*Alb*Cre mouse livers in much weaker level than that in the control mouse livers. The data were analyzed by one-way ANOVA followed by Tukey-Kramer HSD test (*∗P* < 0.05, *∗∗P* < 0.01 and *∗∗∗P* < 0.0001). (D) Models of the KEAP1 status in *Keap1*^*C226S&C613S hetero*^::*Trsp*^*fl/fl*^::*Alb*Cre mouse livers. An intriguing question related to the heterozygous mice is whether heterodimer of the wildtype and KEAP1-Cys226/613 mutant molecules is functional to sense oxidative stress induced by selenoprotein deficiency or not. It is assumed that 25% of KEAP1^C226S&C613S^ homodimer (top panel), 25% of KEAP1^WT^ homodimer (middle panel) and 50% of KEAP1^WT^-KEAP1^C226S&C613S^ heterodimer (bottom panel) consist in the *Keap1*^*C226S&C613S hetero*^::*Trsp*^*fl/fl*^::*Alb*Cre mouse livers. The 25% KEAP1^C226S&C613S^ homodimer retains ubiquitin ligase activity even under the selenoprotein deficiency. In contrast, the 25% KEAP1^WT^ homodimer loses ubiquitin ligase activity to a certain extent by the selenoprotein deficiency. As for the remaining 50% KEAP1^WT^-KEAP1^C226S&C613S^ heterodimer, there are two hypotheses, “monomer inactivation hypothesis” and “dimer inactivation hypothesis”.

**Heterozygous loss of KEAP1-Cys226/613 affects the NRF2-mediated compensation in selenoprotein-deficient liver**. As homozygous loss of KEAP1-Cys226/613 led to poor survival of hepatocyte-specific selenoprotein-deficient mice, we decided to investigate whether heterozygous loss of KEAP1-Cys226/613 affects maintenance of homeostasis of the mice. To this end, we generated *Trsp*^*fl/fl*^::*Alb*Cre in the *Keap1*^*C226S&C613S/+*^ background (referred to hereafter as *Keap1*^*C226S&C613S hetero*^::*Trsp*^*fl/fl*^::*Alb*Cre) mice and examined survival of the mice. As shown in Fig. 7A, the cohort of *Keap1*^*C226S&C613S hetero*^::*Trsp*^*fl/fl*^::*Alb*Cre mice (n = 125) displayed markedly poor survival compared with control *Keap1*^*C226S&C613S hetero*^::*Trsp*^*fl/fl*^ mice (n = 114). The survival curve appeared to be almost the same as that of *Keap1*^*C226S&C613S h^om^o*^::*Trsp*^*fl/fl*^::*Alb*Cre mice (Fig. 6A). Almost 90% of the *Keap1*^*C226S&C613S hetero*^::*Trsp*^*fl/fl*^::*Alb*Cre mice died by 12 weeks after birth and again the death occurred abruptly. Of note, *Trsp*^*fl/fl*^::*Alb*Cre mice showed relatively low mortality frequency, indicating that heterozygous loss of KEAP1-Cys226/613 affects the survival of the hepatocyte-specific selenoprotein-deficient mice.

We surmise that *Keap1*^*C226S&C613S hetero*^::*Trsp*^*fl/fl*^::*Alb*Cre mice die abruptly due to the insufficient NRF2 activation for compensating selenoprotein deficiency. To test this hypothesis, we examined expression levels of NRF2 target genes *Nqo1* and *Gstm1* in *Keap1*^*C226S&C613S hetero*^::*Trsp*^*fl/fl*^::*Alb*Cre mouse livers. It should be noted that the inductions of *Nqo1* and *Gstm1* expressions in these mouse livers were significantly decreased compared to those in *Trsp*^*fl/fl*^::*Alb*Cre mouse livers (Fig. 7B and C), but there remain moderate expressions compared with those of *Keap1*^*C226S&C613S homo*^::*Trsp*^*fl/fl*^::*Alb*Cre mouse livers (see Fig. 1D and E). Statistical analysis of the data shows significant inductions of the *Nqo1* and *Gstm1* expressions in the *Keap1*^*C226S&C613S hetero*^::*Trsp*^*fl/fl*^::*Alb*Cre mouse livers compared with the *Keap1*^*C226S&C613S homo*^::*Trsp*^*fl/fl*^::*Alb*Cre mouse livers (Fig. S2). These results indicate that the NRF2 activation under selenoprotein deficiency is affected even by the heterozygous loss of KEAP1-Cys226/613.

**KEAP1^WT^-KEAP1^C226S&C613S^ heterodimer retains oxidative stress sensor activity**. While the induction of *Nqo1* and *Gstm1* gene expression in *Keap1*^*C226S&C613S hetero*^::*Trsp*^*fl/fl*^::*Alb*Cre mouse livers is weaker than in *Trsp*^*fl/fl*^::*Alb*Cre mouse livers, transcript levels of these genes are still induced by approximately 2-fold relative to the control *Keap1*^*C226S&C613S hetero*^::*Trsp*^*fl/fl*^ mouse livers without *Alb*Cre recombination (Fig. 7B and C). These results indicate that NRF2 is induced in *Keap1*^*C226S&C613S hetero*^::*Trsp*^*fl/fl*^::*Alb*Cre mouse livers, albeit at much lower level than that in the control mice.

In light of this result, we postulated that the ability of KEAP1^WT^ and KEAP1^C226S&C613S^ proteins to form mixed heterodimers in *Keap1*^*C226S&C613S hetero*^::*Trsp*^*fl/fl*^::*Alb*Cre mice implies that this model system can be used to provide a unique insight into KEAP1’s sensitivity to molecular stressors (Fig. 7D). Specifically, as KEAP1^WT^ forms a dimer, and both members of the dimer contain stress sensors, it is currently unknown whether sensor engagement by stress molecules on both members of the dimer is required for KEAP1 inactivation and NRF2 stabilization, or whether sensor engagement by stress molecules on a single member of the dimer is sufficient to inactivate the full KEAP1 dimer complex. These two possibilities can be summarized in the form of the “monomer inactivation hypothesis”, in which inactivation of a single monomer of the KEAP1 dimer is sufficient to inactivate the KEAP1 dimer, and the “dimer inactivation hypothesis”, in which the sensors in both members of the KEAP1 dimer must be bound and inactivated by molecular stressors in order to inhibit KEAP1’s E3 ubiquitin ligase functionality. These contrasting models have differing implications for KEAP1’s sensitivity to oxidative and electrophilic stress, as in the case of the “monomer inactivation hypothesis”, KEAP1 would be much more sensitive to small molecule stressors relative to the “dimer inactivation hypothesis”.

**Fig. 8:**
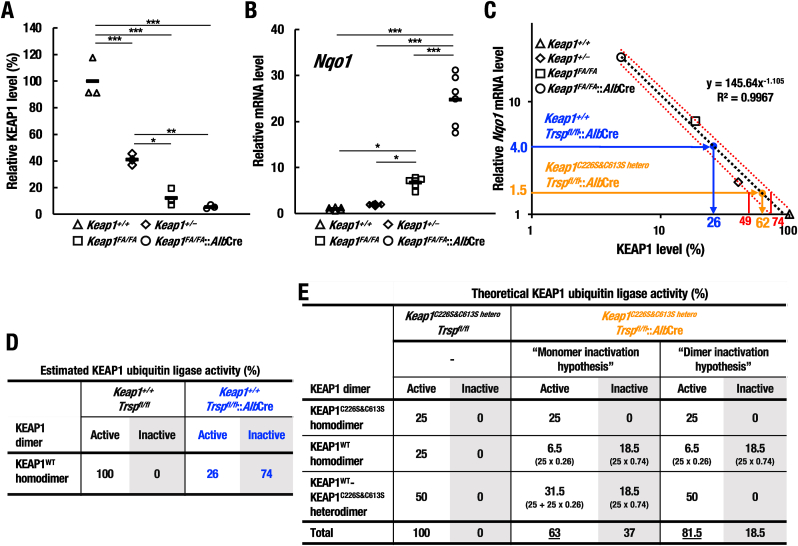
The KEAP1^WT^-KEAP1^C226S&C613S^ heterodimer retains oxidative stress sensor activity. (A) Relative KEAP1 protein levels were quantified and normalized by the band intensity of TUBULIN shown in Supplementary Fig. 3 (n = 3, each). Note that the relative KEAP1 levels of *Keap1*^*+/+*^, *Keap1*^*+/−*^, *Keap1*^*FA/FA*^ and *Keap1*^*FA/FA*^::*Alb*Cre mouse livers were 100, 40, 19 and 5%, respectively. (B) Relative mRNA levels of *Nqo1* in livers of *Keap1^+/+^*, *Keap1*^*+/−*^, *Keap1*^*FA/FA*^ and *Keap1*^*FA/FA*^::*Alb*Cre mice (n = 6, each) at 20 days of age. Relative mRNA levels were analyzed by RT-qPCR. Note that the mRNA levels in these mice showed an inverse correlation to the relative KEAP1 levels. The data were analyzed by one-way ANOVA followed by Tukey-Kramer HSD test (*∗P* < 0.05, *∗∗P* < 0.01 and *∗∗∗P* < 0.0001). (C) Calibration curve of relationship between graded KEAP1 expression levels and relative *Nqo1* mRNA levels in livers of *Keap1*^*+/+*^, *Keap1*^*+/−*^, *Keap1*^*FA/FA*^ and *Keap1*^*FA/FA*^::*Alb*Cre mice based on the data shown in (A, B). Blue and orange arrows indicate estimated KEAP1 activity in *Keap1*^*+/+*^::*Trsp*^*fl/fl*^::*Alb*Cre and *Keap1*^*C226S&C613S hetero*^::*Trsp*^*fl/fl*^::*Alb*Cre mouse livers, respectively. Red dot lines indicate confidence intervals. (D) Estimated KEAP1 ubiquitin ligase activity in the *Keap1*^*+/+*^::*Trsp*^*fl/fl*^ and *Keap1*^*+/+*^::*Trsp*^*fl/fl*^::*Alb*Cre mouse livers based on the *Nqo1* mRNA levels in Fig. 7B. Note that while 100% of KEAP1 is active in *Keap1*^*+/+*^::*Trsp*^*fl/fl*^ mouse livers, active KEAP1 level is estimated approximately 26% in *Keap1*^*+/+*^::*Trsp*^*fl/fl*^::*Alb*Cre mouse livers, indicating that 74% of KEAP1^WT^ homodimers are inactivated by the selenoprotein deficiency. (E) Theoretical KEAP1 ubiquitin ligase activity in *Keap1*^*C226S&C613S hetero*^::*Trsp*^*fl/fl*^ and *Keap1*^*C226S&C613S hetero*^::*Trsp*^*fl/fl*^::*Alb*Cre mouse livers based on the *Nqo1* mRNA levels. Note that there are two hypotheses on the oxidative stress sensing by KEAP1. “Monomer inactivation hypothesis” is that oxidative modification of one KEAP1 molecule in the dimer is sufficient to inactivate KEAP1 ubiquitin ligase activity. “Dimer inactivation hypothesis” is that oxidative modification of both KEAP1 molecules in the dimer is needed to inactivate KEAP1 ubiquitin ligase activity. If modification of only one-molecule of the KEAP1 dimer is sufficient for the inactivation of KEAP1 ubiquitin ligase activity (i.e., the “monomer inactivation hypothesis”), the ubiquitin ligase activity of the KEAP1^WT^-KEAP1^C226S&C613S^ heterodimer should be inactive. On the contrary, if modification of both molecules of the KEAP1 homodimer is required for the inactivation of KEAP1 ubiquitin ligase activity (i.e., the “dimer inactivation hypothesis”), KEAP1^WT^-KEAP1^C226S&C613S^ heterodimer should retain active ubiquitin ligase activity even under the selenoprotein deficiency. The result shows that active KEAP1 level in *Keap1*^*C226S&C613S hetero*^::*Trsp*^*fl/fl*^::*Alb*Cre mouse livers is close to the value for the “monomer inactivation hypothesis”, supporting the notion that oxidative modification of one KEAP1 molecule in the dimer is sufficient to inactivate the KEAP1 ubiquitin ligase activity.

To experimentally test these contrasting models, we generated a calibration curve of the relationship between KEAP1 activity levels and the gene expression level of the NRF2 target gene *Nqo1* (Fig. 8C). Utilizing hypomorphic *Keap1*^*FA*^ allele [39], we generated this curve utilizing data from a series of four genetic mouse models in which KEAP1 protein levels follow a graded distribution from 100% in WT mice down to 5% in *Keap1*^*FA/FA*^::*Alb*Cre mice (Fig. 8A, B and Fig. S3). The expression levels of the NRF2 target genes *Nqo1* (Fig. 8B) and *Gstm1* (Fig. S4A) showed an inverse correlation to KEAP1 protein levels, which is consistent with our previous study [39], and clearly demonstrates that NRF2 transcriptional activity is highly responsive to changes in KEAP1 levels.

Using this calibration curve, the experimentally observed *Nqo1* expression level of 4.0 suggests that the total functional KEAP1 activity level is 26% in *Keap1*^*+/+*^::*Trsp*^*fl/fl*^::*Alb*Cre mice (Fig. 8C). This means that the remaining 74% of the cellular pool of KEAP1 is inactivated by the *Trsp* knockout. For comparison, in WT (*Keap1*^*+/+*^::*Trsp*^*fl/fl*^) mice, KEAP1’s activity was set to 100%, as the mice were experiencing basal, and therefore unstressed, conditions (Fig. 8D).

We next examined KEAP1’s activity in *Keap1*^*C226S&C613S hetero*^::*Trsp*^*fl/fl*^::*Alb*Cre mice, which will comprise a mixed dimer population of 25% KEAP1^C226S&C613S^ homodimers, 25% KEAP1^WT^ homodimers, and 50% KEAP1^WT^-KEAP1^C226S&C613S^ heterodimers, due to the random formation of dimers between the protein products of the two *Keap1* genes (Fig. 8E). As the KEAP1^C226S&C613S^ homodimers are insensitive to selenoprotein deficiency, they will maintain all of their E3 ubiquitin ligase activity in the context of *Trsp* knockout. As established above, 26% of the KEAP1^WT^ homodimers will retain their E3 ubiquitin ligase activity in *Trsp*^*fl/fl*^::*Alb*Cre mice, and as these dimers represent 25% of the total population, they will contribute 6.5% of KEAP1 activity (26% of 25%). Therefore, between KEAP1^C226S&C613S^ homodimers and KEAP1^WT^ homodimers, 31.5% of KEAP1’s E3 ubiquitin ligase functionality will be maintained, with any additional cellular KEAP1 activity being contributed by the KEAP1^WT^-KEAP1^C226S&C613S^ heterodimers (Fig. 8E).

In relation to the KEAP1^WT^-KEAP1^C226S&C613S^ heterodimers, if inactivation of a single member of the dimer is sufficient to inactivate the entire complex, as described by the “monomer inactivation hypothesis”, then 37% of the KEAP1 dimers will be inactivated by selenoprotein deficiency (50% of 74%), while 63% will maintain their ubiquitin ligase activity (100%–37%). As these heterodimers constitute 50% of the total cellular pool of KEAP1, they will contribute 31.5% to the total KEAP1 activity (50% of 63%), which will produce a total cellular KEAP1 activity of 63% (25% + 6.5% + 31.5%) (Fig. 8E).

In contrast, if the “dimer inactivation hypothesis” is true, then inactivation of both members of the dimer with be required to inactivate KEAP1’s E3 ubiquitin ligase activity. As within the KEAP1^WT^-KEAP1^C226S&C613S^ heterodimers the KEAP1^C226S&C613S^ component cannot be inactivated by selenoprotein deficiency, this means that all of these heterodimers will maintain their E3 ubiquitin ligase activity, and thus the total cellular activity of KEAP1 will be 81.5% (25% + 6.5% + 50%) (Fig. 8E).

Based on this analysis, we are able to make a specific prediction for each model. If the “monomer inactivation hypothesis” is true, then in *Keap1*^*C226S&C613S hetero*^::*Trsp*^*fl/fl*^::*Alb*Cre mice we would expect to observe 63% of KEAP1 ubiquitin ligase activity, while in the case of the “dimer inactivation hypothesis”, we would expect 81.5% of KEAP1’s activity to be maintained. As in *Keap1*^*C226S&C613S hetero*^::*Trsp*^*fl/fl*^::*Alb*Cre mice, the actual expression level of *Nqo1* is 1.5, this corresponds to a KEAP1 activity level of 62% using the calibration curve (Fig. 8C). These data provide remarkable experimental support for the “monomer inactivation hypothesis”, which predicts 63% KEAP1 activity, which lies within the confidence interval of the observed data (62% ± 13%), while rejecting the “dimer inactivation hypothesis”, which predicts 81.5% and is therefore outside of the confidence limits. Importantly, this model is also independently supported using a calibration curve based on expression of *Gstm1* (Fig. S4). Taken together, our data demonstrate for the first time that oxidative modification of a single molecule of KEAP1 within the KEAP1 dimer is sufficient to inactivate KEAP1’s E3 ubiquitin ligase function, which argues that KEAP1 functions as an extremely sensitive sensor for oxidative stress.

**Fig. 9:**
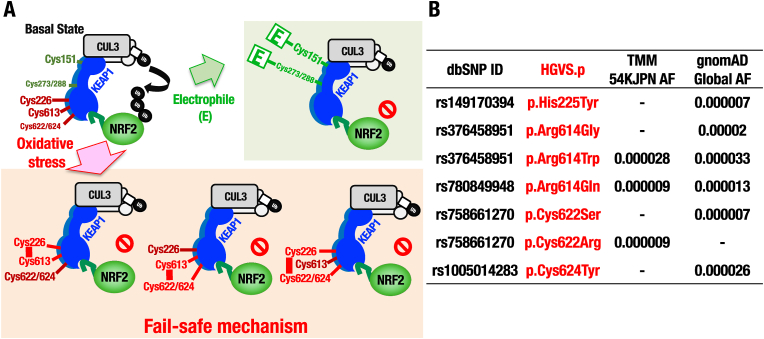
Genetic variants of KEAP1-Cys226/613/622/624 in human. (A) A fail-safe mechanism of KEAP1 for sensing oxidative stresses by Cys226, Cys613, Cys622 and Cys624 in KEAP1. In contrast to the electrophile sensing mechanism, any single mutation to any one of the three parts of the sensor is dispensable for its function, leading to a model in which any combinations of two of the three parts of the sensor are able to form a disulfide bond (Cys226-Cys613, Cys226-Cys622/624 or Cys613-Cys622/624) to function as an oxidative stress sensor. This fail-safe mechanism enables the remaining cysteine residues to rescue the oxidative stress sensor function in the absence of any single part. (B) Genetic variants of Cys226, Cys613, Cys622 and Cys624 or their surrounding amino acid residues in human KEAP1 found from jMorp database, which covers TMM 54KJPN AF (allele frequency) and gnomAD Global AF, are shown.

**Genetic variants of oxidative stress and electrophile sensors in human KEAP1**. Based on the identification of four cysteine residues all contribute to oxidative stress sensor activity in KEAP1, i.e., Cys226, Cys613, Cys622 and Cys624, which together function by forming intramolecular disulfide bonds [24], we proposed the hypothesis that a fail-safe mechanism is utilized by KEAP1 for the sensing of oxidative stress (Fig. 9A). To address the question of whether the proposed mechanism has an impact on oxidative stress sensing in human populations, we investigated genetic variants in the human *KEAP1* gene, focusing especially on the regions in close proximity to the four oxidative stress sensor residues, and thus including the surrounding basic amino acids which also play an important role in the reactivity of sensor cysteine residues [40,41]. To this end, we searched for genetic variants by using the jMorp web database, which was developed by the Tohoku Medical Megabank (TMM) project [25]. In this analysis, whole genome sequencing information, including genetic variants and their allele frequency, from both the TMM 54KJPN panel and gnomAD [26] were analyzed using the jMorp web database.

Through this analysis, we found seven non-synonymous variants in the oxidative stress sensors and their surrounding basic amino acids in the jMorp database (Fig. 9B). Intriguingly, non-synonymous mutations were identified in three of the oxidative stress sensor cysteine residues in KEAP1. Specifically, in the cases of Cys622Ser, Cys622Arg and Cys624Tyr substitution mutants, the cysteine residues are changed to non-thiol residues, which will completely destroy the oxidative stress sensor activity in these motifs. Similarly, in the cases of His225Tyr, Arg614Gly, Arg614Trp and Arg614Gln, the neighboring basic amino acids of the Cys226 and Cys613 sensor residues are substituted with non-basic amino acids, which will function to diminish the reactivity of the sensor cysteine residues in the encoded proteins. The presence of these variants in human populations supports the notion that in the absence of any single oxidative stress sensor motif, the remaining cysteine sensor residues in human KEAP1 can rescue the oxidative stress sensor function by utilizing the fail-safe mechanism.

As described above, Cys151, Cys273 and Cys288 in KEAP1 act as sensors for electrophiles [11,12,23]. In contrast to the oxidative stress sensing mechanism, electrophiles modify the cysteine thiol group through C–S bond formation. Of note, we could not find any variants directly substituting the electrophile sensor cysteines, nor the surrounding basic amino acids adjacent to these cysteine residues in the jMorp database. This observation implies that mutations in the electrophilic sensors cannot be not maintained during molecular evolution, perhaps because of the important electrophile-specific activities of each stress sensor. On the contrary, mutations in the oxidative stress sensor cysteine residues are permissible because of the presence of the fail-safe mechanism, which is based on the combined participation of four cysteine residues, and therefore contains a degree of redundancy. Taken together, we conclude that these seven rare variants within or surrounding Cys226/613/622/624 in KEAP1 further supports the differential use of stress sensors within KEAP1. The fail-safe mechanism makes animals resilient to oxidative stresses, and supports their survival during aerobic respiration, while the electrophilic sensor cysteine residues have acquired their specific and irreplaceable functions during the adaptation to the environment.

## 4 Discussion

**Fig. 10:**
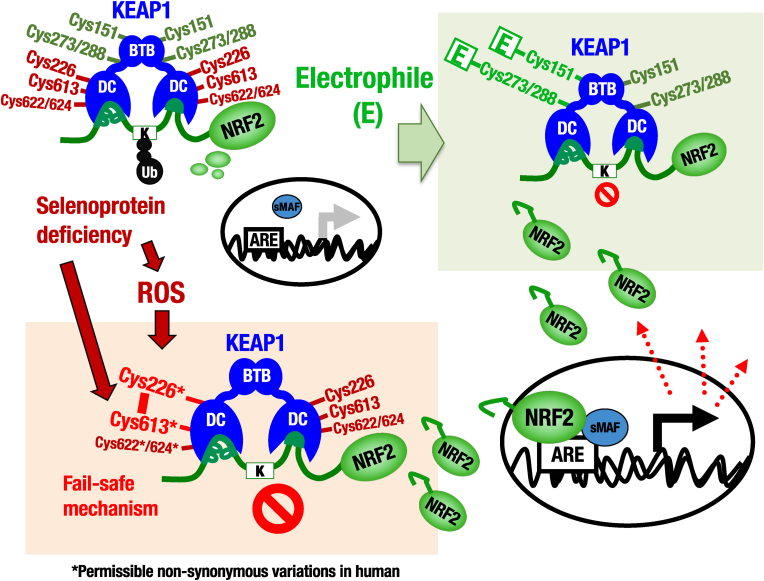
Differential utilization of KEAP1 cysteine sensors for oxidative stresses and electrophiles *in vivo*. *In vivo* mouse analyses have clarified that KEAP1-Cys226/613 and KEAP1-Cys151 are distinctly and specifically utilized for sensing oxidative and electrophile stresses, respectively. In this study, the KEAP1-Cys226/613 pair is shown to be essential as the sensor for the oxidative stresses caused by the selenoprotein deficiency in liver. In contrast, the Cys226/613 pair cannot work as the sensor for electrophiles, while Cys151 cannot sense oxidative stresses *vice versa*. Of note, this study further revealed that oxidative modification of only one molecule in the KEAP1 homodimer is sufficient for the inhibition of ubiquitin ligase activity, thereby resulting in the NRF2 activation, ensuring elaborate and sensitive sensor activity of the KEAP1-NRF2 system for oxidative stresses. Furthermore, inspections of the human whole genome sequence analyses data in jMorp and gnomAD have identified seven rare non-synonymous variants of the oxidative stress sensor cysteine residues (i.e., Cys226, Cys613, Cys622 or Cys624) and their neighborhood residues, but no non-synonymous variant is found in or nearby the electrophilic sensor cysteine residues (i.e., Cys151, Cys273 or Cys288) in human KEAP1. These results revealed the unique molecular natures underlying the sensor activity for oxidative and electrophilic stresses. KEAP1 equips the fail-safe mechanism for sensing oxidative stresses, while strict conservation during the molecular evolution is required for the electrophile sensor cysteine residues.

In this study we have challenged a series of analyses to clarify how oxidative and electrophilic sensors of KEAP1 are operating *in vivo*. As summarized in Fig. 10, *in vivo* mouse analyses revealed that KEAP1-Cys226/613 and KEAP1-Cys151 are distinctly utilized for sensing oxidative and electrophile stresses, respectively. KEAP1-Cys226/613 is essential for NRF2 activation in selenoprotein deficiency *in vivo*. We also found that the KEAP1 heterodimer harboring mutant KEAP1 lacking the Cys226/613 sensor activity concomitant with wild-type KEAP1 can respond to the selenoprotein deficiency, demonstrating that oxidative modification of a single molecule of KEAP1 within the KEAP1 dimer exerts the oxidative stress sensing activity *in vivo*. Our analyses of the human WGS data identified seven rare non-synonymous variants of the oxidative stress sensor cysteine residues and their neighborhood residues, supporting the notion that these oxidative stress sensor variations are permissible due to the fail-safe nature of the KEAP1 oxidative stress sensors. These results highlight physiological significance of the KEAP1 oxidative stress sensor functions and unique molecular natures underlying the sensor activity.

The Cys226/613 pair of KEAP1 has been identified as a sensor for oxidative stresses through the experiments utilizing cultured cells treated with H_2_O_2_ [24,31,37], whereas physiological importance of this oxidative stress sensor *in vivo* remained unclear. A preceding study using a cell culture system revealed formations of both intramolecular disulfide bond via KEAP1-Cys226/613 and intermolecular disulfide bond via KEAP1-Cys151 when cells were treated with H_2_O_2_ [31]. This study clearly shows that KEAP1-Cys226/613 is essential for the NRF2 activation in selenoprotein-deficient livers, while KEAP1-Cys151 is required for CDDO-Im-mediated activation *in vivo*. Thus, we conclude that the Cys226/613 pair acts as the main sensor for oxidative stress *in vivo*, while Cys151 is the sensor for electrophiles. We believe that similar approach utilizing the KEAP1 oxidative stress sensor-deficient mouse will verify importance the KEAP1 sensor functions in various pathological conditions induced by oxidative and electrophilic stresses, such as ischemia-reperfusion tissue injury and exposure to environmental pollutants, respectively.

This study supports the notion firmly that KEAP1-Cys226/613 is important for NRF2 activation in selenoprotein deficiency. Nonetheless, it remains unclear which types of oxidative stress within cells, for instance H_2_O_2_, lipid peroxides or disulfide formation, inactivate the KEAP1 ubiquitin ligase activity. Loss of the selenoprotein members, such as glutathione metabolizing GPXs or thioredoxin metabolizing TXNRDs, elicits increase of intracellular H_2_O_2_ level [[27], [28], [29]]. In fact, our analyses of selenoprotein-deficient macrophages or hematopoietic linage cells show the increase of intracellular H_2_O_2_ level [28,29]. We interpret that this increase of the H_2_O_2_ level is sensed by KEAP1, which subsequently induces compensatory expression of the NRF2 target genes. By contrast, as selenoprotein GPX4 reduces phospholipid hydroperoxide [42], there exists a possibility that KEAP1 also senses lipid peroxides increased due to the selenoprotein deficiency. Indeed, it has been reported that the NRF2 activity is increased in GPX4-deficient mouse livers [43]. In addition, hepatocyte-specific knockout of TXNRD1, which participates in the reduction of protein disulfides, has been reported also to lead to NRF2 activation [44]. Similarly, Auranofin, an inhibitor of TXNRDs, activates NRF2 depending on the KEAP1-Cys226/613 [24], strongly arguing that TXNRD1 is one of the responsible selenoprotein members for the increase of S–S bond formation under selenoprotein deficiency. Taken together, intramolecular disulfides formation in KEAP1 must be increased not only by direct influence of ROS reactivity but also by impairment of disulfide reducing activity provided by TXNRDs.

We found that heterozygous expression of Cys226/613 mutant markedly affects the inducible expression of NRF2-target genes in the hepatocyte-specific selenoprotein-deficient mouse livers. This indicates that even heterozygous loss of functional KEAP1 oxidative stress sensor activity substantially affects the sensor activity and eventually the NRF2-mediated stress response. Thus, the KEAP1 sensor activity retains haploinsufficiency under the *Trsp* deficiency. The human WGS analysis identified seven rare variations in the residues contributing the oxidative stress sensor activity, indicating that the variations in the oxidative stress sensor cysteine residues are permissible because of the presence of the fail-safe mechanism based on the participation of four cysteine residues. By contrast, we did not find any concomitant substitutions of two of these oxidative stress sensor residues, which results in the entire loss of the oxidative stress sensor activity. Our human genome analysis strongly argues that the KEAP1 oxidative stress sensor activity is crucial for animals’ survival and adaptation to the environment.

While KEAP1 acts as a homodimer in the ubiquitin ligase complex [45], this study revealed that S–S bond formation between the cysteine residues in one molecule within the KEAP1 homodimer is sufficient for the inactivation of KEAP1 ubiquitin ligase activity by oxidative stress. We surmise that this mechanism supports elaborate and sensitive sensor machinery of the KEAP1-NRF2 system for oxidative stress. As noted above, whereas heterozygous expression of Cys226/613 mutant affects substantially the inducible expression of NRF2-target genes, there remains considerable level of NRF2-target gene expressions in the heterozygous mouse tissues. S–S bond formation between the cysteine residues in a single molecule of KEAP1 within the KEAP1 dimer can provoke inhibition of the KEAP1 ubiquitin ligase activity, indicating that this ensures the elaborate and meticulous oxidative stress sensor mechanism of KEAP1 in physiological and pathological conditions. Supporting our present results, the alteration of a single molecule in the KEAP1 homodimer has been shown to cause KEAP1 inactivation in a dominant-negative manner in cultured cells, zebrafish, and mice [[46], [47], [48]]. Thus, the KEAP1 dimer equips quite sensitive sensor machinery for oxidative and electrophilic stresses in which modification of a single molecule within the KEAP1 homodimer is sufficient to affect the ubiquitin ligase activity of the KEAP1 dimer complex.

## CRediT authorship contribution statement

**Miu Sato:** Writing – review & editing, Writing – original draft, Investigation, Formal analysis, Data curation, Conceptualization. **Nahoko Yaguchi:** Investigation, Data curation, Conceptualization. **Takuya Iijima:** Investigation, Data curation. **Aki Muramatsu:** Investigation, Conceptualization. **Liam Baird:** Writing – review & editing. **Takafumi Suzuki:** Writing – review & editing, Writing – original draft, Supervision, Project administration, Investigation, Funding acquisition, Conceptualization. **Masayuki Yamamoto:** Writing – review & editing, Writing – original draft, Supervision, Resources, Investigation, Funding acquisition, Conceptualization.

## Declaration of competing interest

None declared.

## Data Availability

We have shared the code of the RNA-seq data generated during this study in GEO repository in the Methods section.
