# Supplementary material for: Sensor systems of KEAP1 uniquely detecting oxidative and electrophilic stresses separately In vivo
Source: Redox Biol. 2024 Sep 17;77:103355. doi: 10.1016/j.redox.2024.103355 (PMC11447412; doi:10.1016/j.redox.2024.103355)
Supplement: Multimedia component 1 [file mmc1.pdf]

Supplementary Figure 1

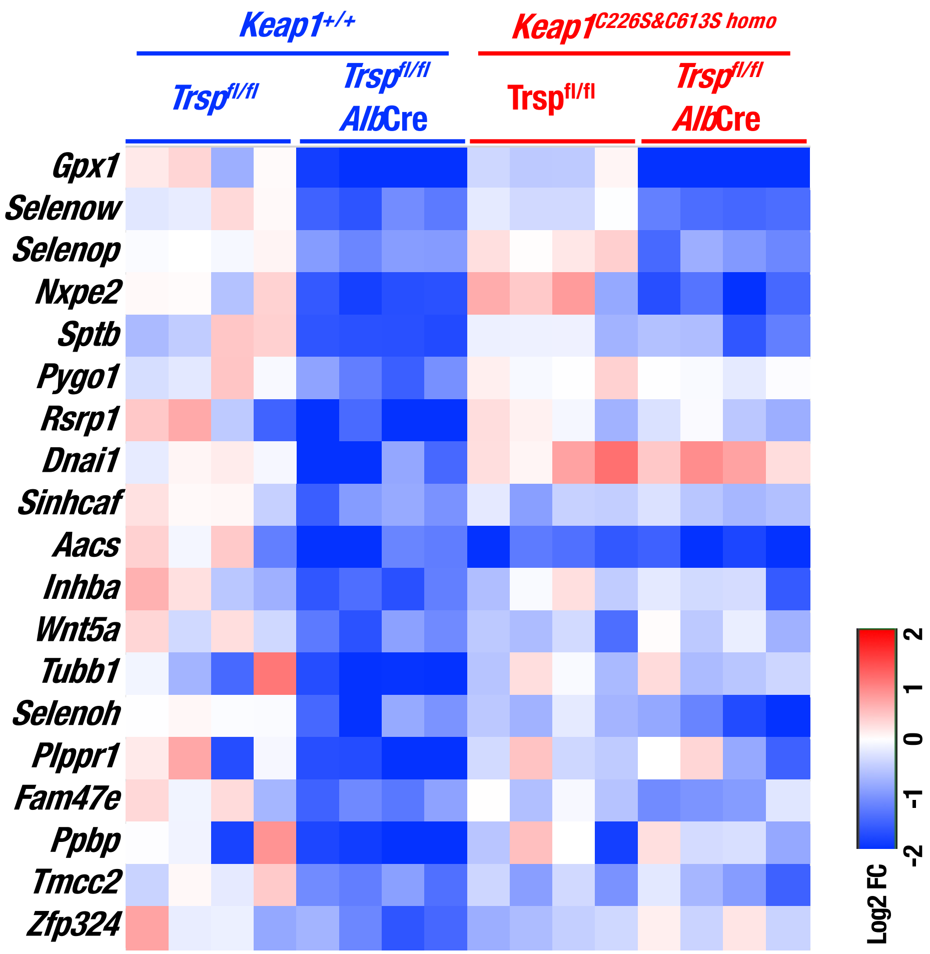

Supplementary Figure 1. Heatmap presentation of the gene expressions down-regulated in *Trsp*$^{fl/fl}$::*Alb*Cre mouse livers compared with those of *Trsp*$^{fl/fl}$ mouse livers. Note that expressions of selenoprotein members including *Gpx1*, *Selenow* and *Selenoh* are all down-regulated in both *Trsp*$^{fl/fl}$::*Alb*Cre mouse livers and *Keap1*$^{C226S\&C613S\ homo}$::*Trsp*$^{fl/fl}$::*Alb*Cre mouse livers, confirming the *Trsp* deletion in both genotype mice.

Supplementary Figure 2

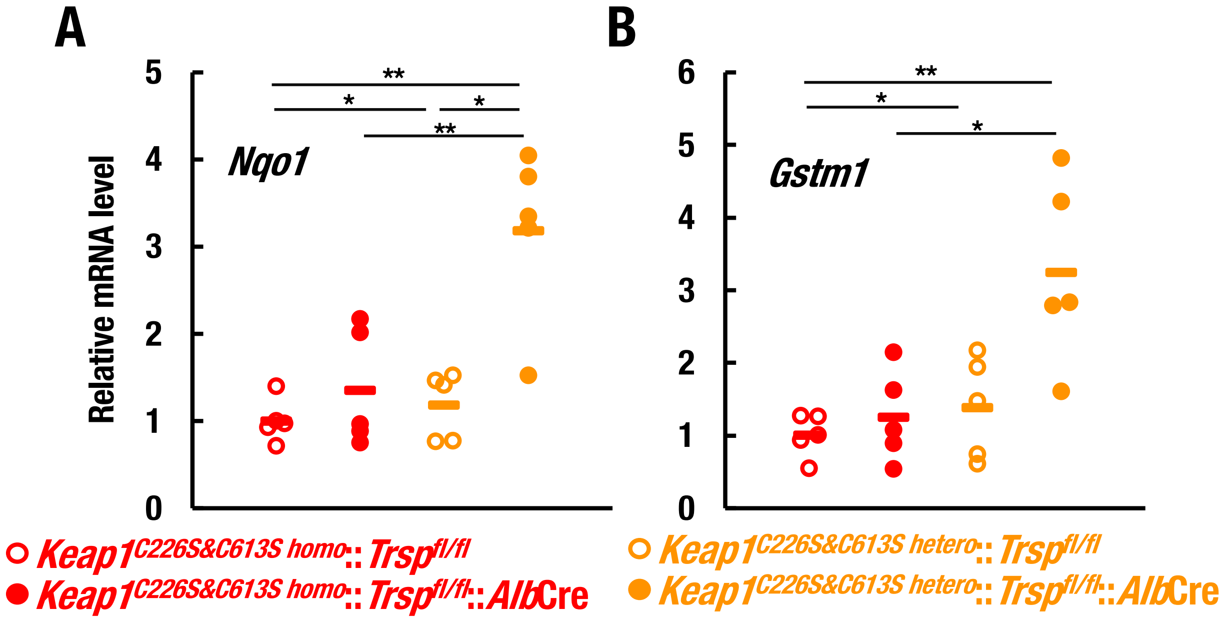

Supplementary Figure 2. Significant induction of the *Nqo1* and *Gstm1* gene expression in the *Keap1*$^{C226S\&C613S\ hetero}$::*Trsp*$^{fl/fl}$::*Alb*Cre mouse livers compared with the *Keap1*$^{C226S\&C613S\ homo}$::*Trsp*$^{fl/fl}$::*Alb*Cre mouse livers. Relative mRNA levels of *Nqo1* (**A**) and *Gstm1* (**B**) in livers of *Keap1*$^{C226S\&C613S\ hetero}$::*Trsp*$^{fl/fl}$, *Keap1*$^{C226S\&C613S\ hetero}$::*Trsp*$^{fl/fl}$::*Alb*Cre (shown in Figure 7B and C), *Keap1*$^{C226S\&C613S\ homo}$::*Trsp*$^{fl/fl}$ and *Keap1*$^{C226S\&C613S\ homo}$::*Trsp*$^{fl/fl}$::*Alb*Cre (shown in Figure 1D and E) mice at 20 days of age were analyzed by RT-qPCR (n=5, each). Data were analyzed by one-way ANOVA followed by Tukey-Kramer HSD test (*P<0.05 and **P<0.01).

Supplementary Figure 3

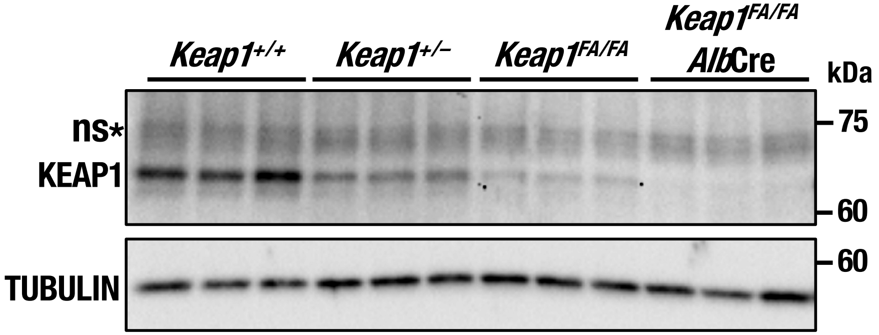

Supplementary Figure 3. KEAP1 protein levels follow a graded distribution from 100% in WT mice down to 5% in *Keap1$^{FA/FA}$*::*Alb*Cre mice . KEAP1 protein levels in livers of *Keap1$^{+/+}$*, *Keap1$^{+/–}$*, *Keap1$^{FA/FA}$* and *Keap1$^{FA/FA}$*::*Alb*Cre mice at 20 days of age. KEAP1 protein levels were analyzed by Western blot. ns stands for a non-specific band.

Supplementary Figure 4

A

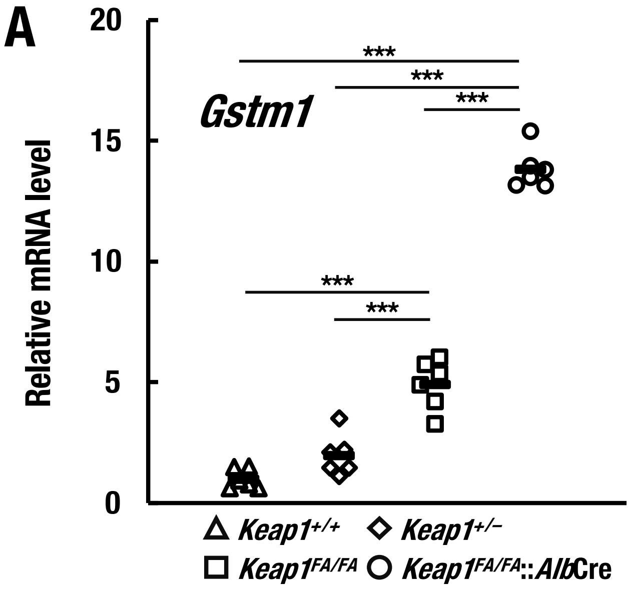

B

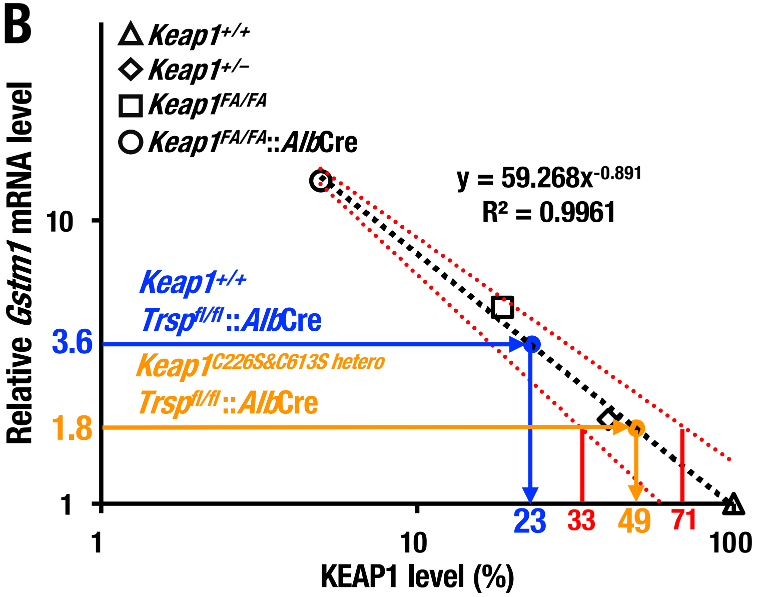

C

Estimated KEAP1 ubiquitin ligase activity (%)

	Keap1$^{+/+}$ *Trsp*$^{fl/fl}$		*Keap1*$^{+/+}$ *Trsp*$^{fl/fl}$::*Alb*Cre	
KEAP1 dimer	Active	Inactive	Active	Inactive
KEAP1^{WT} homodimer	100	0	23	77

D

Theoretical KEAP1 ubiquitin ligase activity (%)

	Keap1$^{C226S\&C613S\ hetero}$ *Trsp*$^{fl/fl}$		*Keap1*$^{C226S\&C613S\ hetero}$ *Trsp*$^{fl/fl}$::*Alb*Cre			
	-		"Monomer inactivation hypothesis"		"Dimer inactivation hypothesis"	
KEAP1 dimer	Active	Inactive	Active	Inactive	Active	Inactive
KEAP1$^{C226S\&C613S}$ homodimer	25	0	25	0	25	0
KEAP1^{WT} homodimer	25	0	5.75 (25 x 0.23)	19.25 (25 x 0.77)	5.75 (25 x 0.23)	19.25 (25 x 0.77)
KEAP1^{WT}-KEAP1$^{C226S\&C613S}$ heterodimer	50	0	30.75 (25 + 25 x 0.23)	19.25 (25 x 0.77)	50	0
Total	100	0	<u>61.5</u>	38.5	<u>80.75</u>	19.25

Supplementary Figure 4. Analysis of the sensor activity in the KEAP1^{WT}-KEAP1$^{C226S\&C613S}$ heterodimer utilizing *Gstm1* mRNA level. (**A**) Relative mRNA levels of *Gstm1* in livers of *Keap1*$^{+/+}$, *Keap1*$^{+/–}$, *Keap1*$^{FA/FA}$ and *Keap1*$^{FA/FA}$::*Alb*Cre mice (n=6, each) at 20 days of age. Relative mRNA levels were analyzed by RT-qPCR. Note that the mRNA levels in these mice showed an inverse correlation to the relative KEAP1 levels. Data were analyzed by one-way ANOVA followed by Tukey-Kramer HSD test (***P<0.0001). (**B**) Calibration curve of relationship between graded KEAP1 protein levels and relative *Gstm1* mRNA levels in livers of *Keap1*$^{+/+}$, *Keap1*$^{+/–}$, *Keap1*$^{FA/FA}$ and *Keap1*$^{FA/FA}$::*Alb*Cre mice. Blue and orange arrows indicate estimated KEAP1 activity in *Keap1*$^{+/+}$::*Trsp*$^{fl/fl}$::*Alb*Cre and *Keap1*$^{C226S\&C613S\ hetero}$::*Trsp*$^{fl/fl}$::*Alb*Cre mouse livers, respectively. Red dot lines indicate confidence intervals. (**C**) Estimated KEAP1 ubiquitin ligase activity in the *Keap1*$^{+/+}$::*Trsp*$^{fl/fl}$ and *Keap1*$^{+/+}$::*Trsp*$^{fl/fl}$::*Alb*Cre mouse livers based on the *Gstm1* mRNA levels in Figure 7C. Note that while 100% of KEAP1 is active in *Keap1*$^{+/+}$::*Trsp*$^{fl/fl}$ mouse livers, functional KEAP1 activity level is estimated approximately 23% in *Keap1*$^{+/+}$::*Trsp*$^{fl/fl}$::*Alb*Cre mouse livers, indicating that 77% of KEAP1^{WT} homodimers are inactivated by selenoprotein deficiency. (**D**) Theoretical KEAP1 ubiquitin ligase activity in *Keap1*$^{C226S\&C613S\ hetero}$::*Trsp*$^{fl/fl}$ and *Keap1*$^{C226S\&C613S\ hetero}$::*Trsp*$^{fl/fl}$::*Alb*Cre mouse livers based on the *Gstm1* mRNA levels.
