# Supplementary material for: Sensor systems of KEAP1 uniquely detecting oxidative and electrophilic stresses separately In vivo
Source: Redox Biol. 2024 Sep 17;77:103355. doi: 10.1016/j.redox.2024.103355 (PMC11447412; doi:10.1016/j.redox.2024.103355)
Supplement: Multimedia component 2 [file mmc2.docx]

**Supplementary Table 1**

| Oligo | Sequence | Experiment |
| --- | --- | --- |
| Cre1 | TGCCACGACCAAGTGACAGCAATG | PCR |
| Cre2 | AGAGACGGAAATCCATCGCTCG | PCR |
| SeCond F1 | GGCTGACTTACAGTTTCAGAGGCAC | PCR |
| SeCond R1 | ACCAACTCCCCTTGAGTTTAGACGC | PCR |
| Keap1 d132 | CGGGATCCCCATGGAAAGGCTTATTGAGTTC | PCR |
| TVneo | TCAGAGCAGCCGATTGTCTGTTGTGCCCAGTCAT | PCR |
| Keap1 intR | CAGTTTTCCTCCAGCCTGTC | PCR |
| Keap1 720s | CAGCAGTTAAGGGCACCAATGC | PCR |
| Keap1 925as | CCTGCCTCAGCTTCCCATCA | PCR |
| Keap1 CKOg2 | TCGGCATGGACGAGCTGTAC | PCR |
| C226S-F | CCAAGCAGGAGGAGTTCTTCAAC | TaqMan |
| C226S-R | GCAGCGTACGTTCAGATCATC | TaqMan |
| C226WT-P | VIC-TGTCACACTGCCAGCTG-MGB | TaqMan |
| C226S-P | FAM-CTGTCACACTCCCAGCTG-MGB | TaqMan |
| C613S-F | GTGAGGTGACCCGCATGAC | TaqMan |
| C613S-R | TTCAGCAGGTACAGTTTTGTTGATC | TaqMan |
| C613WT-P | VIC-ATGGAACCCTGTCGGAA-MGB | TaqMan |
| C613S-P | FAM-ATGGAACCCTCCCGGAA-MGB | TaqMan |
| C151S-F | AAGGCTTATTGAGTTCGCCTACA | TaqMan |
| C151S-R | CCACGCTGTCAATCTGGTACAT | TaqMan |
| C151WT-P | FAM-CAGGACACACTTCT-MGB | TaqMan |
| C151S-P | VIC-CAGGACCGACTTC-MGB | TaqMan |
