# Supplementary material for: Sensor systems of KEAP1 uniquely detecting oxidative and electrophilic stresses separately In vivo
Source: Redox Biol. 2024 Sep 17;77:103355. doi: 10.1016/j.redox.2024.103355 (PMC11447412; doi:10.1016/j.redox.2024.103355)
Supplement: Multimedia component 3 [file mmc3.docx]

**Supplementary Table 2**

| Oligo | Sequence |
| --- | --- |
| mHprt-F | CTGGTGAAAAGGACCTCTCG |
| mHprt-R | TGAAGTACTCATTATAGTCAAGGG |
| mHprt-P | FAM-ATCCAACAAAGTCTGGCCTGTATCCAAC-TAMRA |
| mNqo1-F | AGCTGGAAGCTGCAGACCTG |
| mNqo1-R | CCTTTCAGAATGGCTGGCA |
| mNqo1-P | FAM-ATTTCAGTTCCCATTGCAGTGGTTTGGG-TAMRA |
| mGstm1-F | CCTATGATACTGGGATACTGGAACG |
| mGstm1-R | GGAGCGTCACCCATGGTG |
| mGstm1-P | FAM-CGCGGACTGACACACCCGATCC-TAMRA |
| mGsta4-F | GGGAACAGTATGAGAAGAAGATGCAAAA |
| mGsta4-R | CCCATCGATTTCAACCAAGG |
| mGsta4-P | FAM-TGCACACCTGCTTTTCGGCCAAG-TAMRA |
| mGclc-F | ATCTGCAAAGGCGGCAAC |
| mGclc-R | ACTCCTCTGCAGCTGGCTC |
| mGclc-P | FAM-ACGGGTGCAGCAAGGCCCA-TAMRA |
| mTxnrd1-F | AGAAAGTGCTGGTCTTGGATTTTG |
| mTxnrd1-R | TCTGGTCCCAAGAGGAGTCGGTGTG |
| mTxnrd1-P | FAM-TCTGGTCCCAAGAGGAGTCGGTGTG-TAMRA |
| mTxn1-F | CATTGCCTGTTCTTGCAGAGG |
| mTxn1-R | CCCTGGAACTGGAGGAACAA |
| mTxn1-P | FAM-TCAGTTCTCAGCATCCATACGGCAGC-TAMRA |
